# Acetazolamide potentiates the anti-tumor potential of HDACi, MS-275, in neuroblastoma

**DOI:** 10.1186/s12885-017-3126-7

**Published:** 2017-02-24

**Authors:** Reza Bayat Mokhtari, Narges Baluch, Micky Ka Hon Tsui, Sushil Kumar, Tina S. Homayouni, Karen Aitken, Bikul Das, Sylvain Baruchel, Herman Yeger

**Affiliations:** 1 Developmental and Stem Cell Biology, The Hospital for Sick Children, Toronto, ON Canada; 2 Department of Paediatric Laboratory Medicine, The Hospital for Sick Children, Toronto, ON Canada; 3 Institute of Medical Science, University of Toronto, Toronto, ON Canada; 4 Department of Paediatrics, Division of Hematology/Oncology, The Hospital for Sick Children, Toronto, ON Canada; 5 Department of Pathology and Molecular Medicine, Queen’s University, Kingston, ON Canada; 6 Department of Immunology and Infectious Diseases, The Forsyth Institute, Cambridge, MA USA

**Keywords:** Neuroblastoma, Histone deacetylases, Carbonic anhydrases, HDAC inhibitor, Acetazolamide, MS-275

## Abstract

**Background:**

Neuroblastoma (NB), a tumor of the primitive neural crest, despite aggressive treatment portends a poor long-term survival for patients with advanced high stage NB. New treatment strategies are required.

**Methods:**

We investigated coordinated targeting of essential homeostatic regulatory factors involved in cancer progression, histone deacetylases (HDACs) and carbonic anhydrases (CAs).

**Results:**

We evaluated the antitumor potential of the HDAC inhibitor (HDACi), pyridylmethyl-N-{4-[(2-aminophenyl)-carbamoyl]-benzyl}-carbamate (MS-275) in combination with a pan CA inhibitor, acetazolamide (AZ) on NB SH-SY5Y, SK-N-SH and SK-N-BE(2) cells. The key observation was that the combination AZ + MS-275 significantly inhibited growth, induced cell cycle arrest and apoptosis, and reduced migration capacity of NB cell line SH-SY5Y. In addition, this combination significantly inhibited tumor growth in vivo, in a pre-clinical xenograft model. Evidence was obtained for a marked reduction in tumorigenicity and in the expression of mitotic, proliferative, HIF-1α and CAIX. NB xenografts of SH-SY5Y showed a significant increase in apoptosis.

**Conclusion:**

MS-275 alone at nanomolar concentrations significantly reduced the putative cancer stem cell (CSC) fraction of NB cell lines, SH-SY5Y and SK-N-BE(2), in reference to NT2/D1, a teratocarcinoma cell line, exhibiting a strong stem cell like phenotype in vitro. Whereas stemness genes (OCT4, SOX2 and Nanog) were found to be significantly downregulated after MS-275 treatment, this was further enhanced by AZ co-treatment. The significant reduction in initial tumorigenicity and subsequent abrogation upon serial xenografting suggests potential elimination of the NB CSC fraction. The significant potentiation of MS-275 by AZ is a promising therapeutic approach and one amenable for administration to patients given their current clinical utility.

## Background

Neuroblastoma (NB) is a tumor derived from the primitive neural crest that forms the peripheral sympathetic nervous system. Despite aggressive treatment long-term survival for high-risk NB is less than 40%, due mainly to metastasis and relapse [1]. Intensive multimodal therapy has failed to improve long-term survival significantly [1]. Although NB constitutes only 7% of pediatric malignancies, it accounts for more than 10% of mortality from childhood cancer [1]. Therefore, newer treatment strategies are needed to address the therapeutic challenges of this highly aggressive pediatric cancer. As expression of both carbonic anhydrases (CA) and histone deacetylases (HDACs) are reported to be elevated in NB, they represent potential novel therapeutic targets for NB [1–3]. The benzamide class I specific HDAC inhibitor (HDACi), pyridylmethyl-N{4-[(2-aminophenyl)-carbamoyl]-benzyl}-carbamate (MS-275) alone or in combination with other compounds (ex. azacytidine, an inhibitor of DNA methylation), has been in clinical trials for leukemia and other solid tumors [4, 5]. HDACi has been proven to be effective in NB preclinical studies [6]. MS-275 is noted for its potent anti-cancer abilities, long serum half life, and selective HDACi properties [7]. In particular, Jaboin et al. reported that MS-275 induced apoptosis of NB KNCR in vitro after 48 h, and significantly reduced growth of adrenal orthotopic xenografts [8]. MS-275 decreased cell viability and induced differentiation of NB cell lines (BE(2)-C and Kelly) [9, 10]. Other studies have shown synergistic effects of HDACi with some of the conventional chemotherapeutic agents [11].

Maintaining pH homeostasis, as governed by carbonic anhydrases (CAs) [12] is essential for tumor cell survival and progression. One of the 15 CA isoforms, CAIX, is associated with malignant progression and metastasis [12]. CAIX in particular correlates with metastasis and tumor progression, in many cancers including NB [12, 13]. Further, upregulation of HIF1-α in the hypoxic tumor microenvironment upregulates CAIX, its downstream target [12, 14]. This occurs in NB cell lines exposed to chronic hypoxia [13]. In NB patients higher expression of membrane CAIX in NB biopsies is inversely associated with overall survival and event free survival [13]. In addition, higher levels of membrane CAIX are correlated with the less well-differentiated phenotype, MYCN amplification and unfavorable pathology [14]. The critical role of CAs in tumor survival has encouraged research into the efficacy of CA inhibitors against several types of cancer [15].

The pan-CA inhibitor, acetazolamide (AZ), is routinely administered for the treatment of high altitude sickness and glaucoma [16]. We previously reported that AZ reduces cell viability colony formation, and inhibited tumor growth in lung carcinoid and bladder cancer cell lines in a concentration-dependent manner [17]. In these studies AZ potentiated the anti-tumor effect of sulforaphane, an isothiocyanate with HDACi activity. In human renal carcinoma and cervical cancer cells, AZ and AZ-based derivatives, as single agent or in combination therapy with synthesized aromatic sulfonamides with high affinity for CAIX demonstrated antitumor activity including inhibition of cell proliferation, induction of apoptosis and suppression of tumor cell invasiveness [18, 19].

More recent evidence suggests that combining a carbonic anhydrase inhibitor with a HDACi might indeed be more effective than either agent alone since they target different steps in the response of tumor cells to hypoxia prevalent in almost all cancers [17, 20]. In fact, the hypoxic microenvironment positively enhances expansion of cancer stem cells (CSCs) where upregulation of HIF1-α drives expression of CAIX associated with CSC expansion [21, 22]. Further, MS-275 can increase senescence in mesenchymal stem cells, and decreases expression of stemness genes (e.g. Sall-4 and BMI-1) [23]. Therefore, we postulated that combining AZ with MS-275, a potent selective HDACi, would be more effective than either single agent alone against NB. MS-275 at low μM concentrations has previously been shown to negatively affect NB cell viability in vitro [8]. We confirmed this observation and provide evidence of the ability of AZ to significantly potentiate the MS-275 HDACi effect on NB in vitro and in vivo.

## Methods

### Materials

Acetazolamide (AZ), cisplatin (CDDP), dimethyl sulfoxide (DMSO) and MS-275 were obtained from Sigma-Aldrich (Oakville, ON, Canada). Culture media, AMEM, DMEM/F12 and DMEM, and supplements, fetal bovine serum (FBS) and penicillin-streptomycin, were purchased from Gibco (Burlington, ON, Canada). Bovine serum albumin (BSA) was obtained from Invitrogen (Grand Island, NY, USA). Matrigel was purchased from BD Biosciences company (La Jolla, CA, USA). Methylcellulose was obtained from StemCell Technologies (Vancouver, BC, Canada) and phosphate buffered saline (PBS) from Multicell (St. Bruno, QC, Canada).

### Cell lines

Cells were purchased from the American Type Culture Collection (ATCC) as follow: N-type, MYCN non-amplified SH-SY5Y (CRL-2266) and SK-N-SH (HTB-11) and MYCN amplified, SK-N-BE(2) (CRL-2271), and teratocarcinoma NT2/D1 (CRL-1973). Cells were cultured in AMEM (SH-SY5Y and SK-N-BE(2)) and DMEM/F12 (NT2/D1) supplemented with 10% fetal bovine serum and 1% penicillin/streptomycin (Multicell, St. Bruno, Quebec) at 37 °C in a humidified atmosphere of 5% CO_2_. Reference normal neuronal stem cell strains (NSC6539 and NSC6562) were kindly provided by Dr. Peter Dirks (SickKids) and maintained in Neurocult and Neuronal stem cell Expansion media-Human from Stemcell Technologies (Stemcell Technologies,Vancouver, BC, Canada) supplemented with 2 mM L-Glutamine, 75 μg/ml BSA, 10 ng/ml (B27, EGF and FGF), 2 μM/ml heparin and 1% penicillin/streptomycin (Multicell, St. Bruno, Quebec, Canada). The cells were grown on poly-L-ornithine and laminin (Sigma-Aldrich, Oakville, ON, Canada) coated plates at 37 °C and 5% CO_2_. The cells were fed every 3–4 days.

### Trypan blue exclusion assay

Standard procedures were performed as described [17]. Briefly, cell viability was assessed by trypan blue exclusion assay by observing the number of trypan blue positive cells versus total cells counter per microscopic field.

### AlamarBlue cytotoxicity assay

Standard protocol was performed as described [17]. Percent survival vs. control (DMSO- 0.2x10^−4^μM) of cells when treated with AZ, MS-275 and AZ + MS-275 were observed using AlamarBlue agent (AbD Serotec, MorphoSys, Raleigh, NC, USA) agent (10% of total volume) was added to each well for 4 h before fluorometric detection. Fluorescence was measured using the SPECTRAmax Gemini Spectrophotometer (excitation 540 nm; emission 590 nm).

### In-cell western assay

10^5^ cells were seeded into 96 well plates and treated for 48 h with 1.5 μM MS-275. Following treatment, cells were washed twice with PBS and fixed with 4% paraformaldehyde for 20 min. Cells were then permeabilized with 0.1% Triton-X 100 for 5 min and washed twice with PBS. Cells were blocked in 1x Odyssey blocking buffer (LI-COR, Guelph, ON, Canada) for 2 h at room temperature. Primary antibodies against p16 (1/50; Santa Cruz, Santa Cruz, CA, USA), p21 (1/20; Santa Cruz, Santa Cruz, CA, USA), p27 (1/20; Santa Cruz, Santa Cruz, CA, USA), BCL-2 (1/100; Cell Signaling Technology, Toronto, ON, Canada), cyclin D1 (1/10; Neomarkers/Lab Vision,ThermoScientific, Fremont, CA, USA), and CDK4 (1/100; Neomarkers/Lab Vision,ThermoScientific, Fremont, CA, USA) were diluted in Odyssey blocking buffer at indicated ratios and added to cells overnight at 4 °C. Pan-actin antibody (1/100; CEDARLANE, Burlington, Ontario, Canada) was also added in conjunction with the other antibodies to serve as a control of cell content. Cells were then washed with a 0.1% Tween 20 (Fisher Scientific Co, Markham, ON, Canada) solution for 5 min and repeated five times. Fluorescently labeled Li-COR secondary antibody (Goat-anti-Rabbit IRDye 680; LI-COR, Guelph, ON, Canada), (Goat-anti-Mouse IRDye 800; LI-COR, Guelph, ON, Canada) were then added at a dilution of 1/500 and cells treated for 1 h at RT. In wells where actin was not used as a cell content control, DRAQ5/Sapphire700 were added at 1 mM and 1/1000 dilution respectively. Cells were then again washed with 0.1% Tween 20 solution five times for 5 min. Cells were imaged on the Odyssey Infrared Imaging System at excitation of 700 nm and 800 nm. Total fluorescence was quantified and adjusted to cell content control of either actin or DRAQ5/Sapphire700.

### Propidium Iodide cell cycle assay

Briefly, 2 × 10^6^ cells treated with AZ and/or MS-275 were lifted by citrate saline and fixed in 80% ice-cold ethanol for 48 h. Cells were then pelleted and re-suspended in 2 mg/mL RNase A (Sigma-Aldrich, Oakville, ON, Canada) for 5 min. A 0.1 mg/mL propidium iodide solution (Sigma-Aldrich, Oakville, ON, Canada) was added, incubated for 30 min at RT, and cells filtered through a cell-strainer into a 5 mL polystyrene tube. Labeled cells were analyzed on a BD FACSCAN flow cytometer. Data was fitted by the Watson-Pragmatic model on FlowJo Software (Tree Star, Ashland, OR, USA).

### Methylcellulose clonogenic assay

Standard protocol was performed as follows [17], cultures were trypsinized and single cells were suspended in methycellulose medium (Methocult; StemCell Technologies, Vancouver, BC, Canada). In this process, 1.2x10^4^ SH-SY5Y, 7.5 x 10^3^ SK-N-BE(2) and 2 × 10^3^ NT2/D1 cells/mL were placed into a 40% methycellulose solution supplemented with 10% FBS, 1% antibiotics and 49% culture medium. MS-275 concentrations ranging from 10nM to 3 μM were added to the methycellulose. Cells in methylcellulose were gently vortexed and distributed into non-adherent 35 mm tissue culture dishes with a blunt end 16 gauge needle. Samples were placed in a 37 °C incubator in 5% CO_2._ After 2 weeks colonies were photographed and counted on a phase contrast microscope using a grading dish. Clonogenicity was determined as the average of number of colonies per dish for each group of cells.

### Side Population (SP) assay

Briefly, 10^6^ cells/ml were lifted with citrate saline (0.05 M) and incubated for 1.5 h with 5 μg/mL Hoechst 33342 (bisbenzimide trihydrochloride); (Sigma-Aldrich, Oakville, ON, Canada) in a 37 °C water bath. Negative controls were prepared by prior addition of 50 μM Verapamil HCl (Sigma-Aldrich, Oakville, ON, Canada), calcium channel blocker. Cells were washed, counterstained with 1 μg/mL propidium iodide (Sigma-Aldrich, Oakville, ON, Canada) and analyzed on a BD LSRII flow cytometric analyzer.

### Flow cytometry for cell surface ABCG2

For flow cytometry, 10^5^ cells/ml were lifted with trypsin and were blocked in cold 5% BSA/PBS solution at 4 °C for 15 min. Next, cells were treated with anti-ABCG2 conjugated to phycoerythrin (R&D Systems, Mineapolis, MN, USA) for 45 min. After being washed three times with cold PBS, cells were resuspended in PBS solution containing 7-AAD (BD Pharmingen, San Jose, CA, USA) and then analyzed on a BD LSRII flow cytometric analyzer. Cells negative for 7-AAD were gated to exclude non-viable cells. Gating was determined from the negative trypsin controls.

### Flow cytometry for OCT4, SOX2, Nanog

Adherent cells were lifted by trypsin, washed and fixed with 4% paraformaldehyde (Canemco, St. Laurent, Quebec, Canada) in PBS. 3 × 10^6^ cells were permeabilized with 0.1% Triton-X in PBS, washed twice with PBS and blocked with 5% BSA/PBS solution for 1 h at RT. Cells were incubated overnight at 4 °C in primary antibody against OCT4 (1/200; Cell Signaling, Danvers, MA, USA), SOX2 (1/200; R&D Systems, Mineapolis, MN, USA) or Nanog (1/200; Cell Signaling Technology, Toronto, ON, Canada) in 5% BSA/PBS. Cells were subsequently washed three times with PBS and incubated with a chicken-anti-rabbit Alexa Fluor-488 (1/3500; Invitrogen, Carlsbad, CA, USA) or goat-anti-mouse R-Phycoerythrin (1/500; Caltag, Burlingame, CA, USA) secondary antibody in 5% BSA/PBS, for 1 h in room temperature. Cells were washed and analyzed on a BD FACSCAN flow cytometer.

### Immunofluorescence labeling

Cells were grown on glass coverslips until 75% confluent and then treated with MS-275. Immunofluorescence was performed [24] with primary antibodies to OCT4 (1/300; Cell Signaling Technology, Toronto, ON, Canada), SOX2 (1/250; R&D Systems, Minneapolis, MN, USA) and Nanog (1/200; Cell Signaling Technology, Toronto, ON, Canada) followed by incubation in AlexaFluor secondary antibodies (Invitrogen, Grand Island, NY, USA), and mounting in PBS/glycerol.

### Western blot analysis

Cells were lysed with RIPA extraction buffer (MBiotech, Seoul, Korea) supplemented with a CompleteMini protease inhibitor tablet (Roche, Indianopolis, IN, USA). 100ug of protein was loaded for SH-SY5Y lysates, and 20 μg for NT2/D1 lysates. OCT4, SOX2 (Cell Signaling Technology, Toronto, ON, Canada) and Nanog (Cell Signaling Technology, Toronto, ON, Canada) antibodies were used at 1/1000 dilution. Secondary horseradish peroxidase conjugated antibodies (Jackson Immunoresearch, West Grove, PA, USA) were used at a dilution of 1/6000 and signal was detected with the Supersignal chemiluminescence detection system (Pierce Biotechnology, Rockford, Il, USA).

### Wound healing assay

SH-SY5Y cells were seeded in a 48-well plate on glass cover slips and allowed to adhere overnight at a density of 10^5^ cells/well in 500 μl culture medium in triplicate. Wells were marked with a straight black line on the bottom for orientation. At the time of 90% confluence, cell monolayers were scratched with a 200 μl pipette tip using the marker guide. Loosened non-adherent cells were washed off with medium. Fresh medium was added to the cultures with additions of AZ (10 μM, 20 μM, 40 μM) and MS-275 (0.75 μM, 1.5 μM and 3 μM) and cultured for 48 h. After the 48 h period cells were washed with PBS and fixed in 4% paraformaldehyde. After three washes in PBS, cells were stained with 1% Crystal violet in 20% methanol. Phase contrast light microscopic images (10x original magnification) were taken at time points of 0, 48 and 72 h of treatment. Migrated cells were counted manually to quantify numbers of cells migrated to wound area using NIH Image J program. Each experiment was conducted three times in triplicate and one representative assay is shown.

### Xenograft studies for determining the in vivo efficacy of AZ, MS-275, and AZ + MS-275 combination

For the in vivo xenograft study, 4–6 weeks-old female NOD/SCID mice were obtained from the animal facility at The Hospital for Sick Children. The animal use protocols were approved by the Animal Care Committee, Sickkids Research Institute. Animals were treated per guidelines of Canadian Council on Animal Care (CCAC). Subcutaneous xenograft tumors were developed by injecting SH-SY5Y cells (2 × 10^6^) into the inguinal fat pad of NOD/SCID mice. When tumor diameter reached 0.5 cm, the mice were randomized into four groups (5 mice per group). The control and treatment groups received intraperitoneal injections of vehicle (PBS) or AZ (40 mg/kg), MS-275 (20 mg/kg) or the combination, respectively, every day for 2 weeks. Experiments were terminated when tumor sizes exceeded 2 cm^3^ in volume or animals showed signs of morbidity. Tumor diameters were measured on a daily basis until termination. The long (D) and short diameters (d) were measured with calipers. Tumor volume (cm^3^) was calculated as V = 0.5 × D × d^2^. After euthanizing the mice, tumors were resected, weighed and fixed in 10% neutral-buffered formalin at room temperature and processed for histopathology. For the in vivo serial heterotransplantation analysis, 2x10^6^ untreated and pretreated AZ + MS-275 cells, manually and enzymatically dissociated from treated tumors, were injected subcutaneously to NOD/SCID mice. Growth rates were measured 2–3 times per week. On the 38th day, the animals were sacrificed, after which tumors were removed and weighed.

### Electron microscopic analysis

Tumor fragments were fixed in 4% formaldehyde and 1% glutaraldehyde in phosphate buffer, pH 7.4, and post fixed in 1% osmium tetroxide. Tumor tissues were then dehydrated in a graded series of acetone from 50 to 100% and subsequently infiltrated and embedded in Epon-Araldite epoxy resin. The processing steps from post fixation to polymerization of resin blocks were carried out in a microwave oven, Pelco BioWave 34770 (Pelco International, Clovis, CA, USA). Ultrathin sections were cut with a diamond knife on the Reichert Ultracut E (Leica Inc., Vienna, Austria). Uranyl acetate and lead citrate were used to stain the sections before being examined in the JEM-1011 (JEOL USA Inc., Peabody, MA, USA). Digital electron micrographs were acquired directly with a 1024 × 1024 pixels CCD camera system (AMT Corp., Danvers, MA, USA) attached to the ETM (1200 EX electron microscope).

### Immunohistochemistry

Standard protocol was performed as described [17]. Immunohistochemistry (IHC) was performed on paraffin sections where slides underwent a series of deparaffinization and rehydration washes which were further processed for antigen retrieval and blockage of endogenous peroxidase activity. Sections were then incubated with secondary antibody broad-spectrum poly horseradish peroxidase, and incubation with DAB. The percentage of positive cells was calculated by using the formula [X (6 low power fields of positive staining)/Y(total count per 6 fields) × 100]. The level of IHC of the positive cells was also examined by ImageJ64 software.

### Terminal deoxynucleotidyl transferase dUTP nick end labelling (TUNEL) analysis

The TUNEL assay was performed on 5 μm sections prepared from formalin-fixed, paraffin-embedded xenografts, using the In Situ Cell Death Detection Kit (Roche, Indianopolis, IN, USA) and the protocol suggested by the manufacturer, except that the positive control was treated with 500 units/ml DNaseI (Roche, Indianopolis, IN, USA) before adding the TUNEL reaction buffer. The peroxidase reaction was carried out with stable DAB solution (Invitrogen, Grand Island, NY, USA). Finally slides were counterstained with haematoxylin and examined under light microscopy.

### Statistical analyses

The data are presented as mean +/− SD. Statistical analysis of variance was run based on triplicate experiments and performed with Graphpad Prism 5.0 software (Hearne Scientific Software, Chicago, IL, USA) using 2-tailed Student’s *t*-test or 2-tailed paired *t*-test. Asterisks denote significance (*) *p* ≤ 0.05; (**) *p* ≤ 0.01; (***) *p* ≤ 0.001. Coefficient of Drug Interaction (CDI) was used for the assessment of drug interaction (antagonistic, synergistic and additive). CDI was calculated by formula AB/AxB; where AB, A and B are the cytotoxicity ratio of the combination, AZ single agent and MS-275 single agent, respectively. Cytotoxicity ratio at a certain drug concentration is the ratio of %viability of cells at that concentration to % viability of untreated cells. CDI equal to 1, < 1 and > 1 is considered as additive, synergistic and antagonistic, respectively.

## Results

### AZ, MS-275 and AZ + MS-275 treatments inhibit growth of NB SH-SY5Y cells

To determine the effect of AZ, MS-275 and AZ + MS-275 treatments on the growth of NB SH-SY5Y, SK-N-SH and SK-N-BE(2) cells, we used the AlamarBlue and trypan blue assays. As reference normal controls, we included the neuronal stem cell strains, NSC6539 and NSC6562, which represent neural lineage derived stem cells albeit from the central nervous system [25]. We chose clinically acceptable concentration ranges for AZ (0-160 μM) [17, 26] and MS-275 (0–3 μM) [7–9, 27]. It should be noted that AlamarBlue also indicates effects on oxidative phosphorylation as it is a substrate for the last step in oxidative phosphorylation. Thus it can also reflect metabolic mitochondrial effects.

**Fig. 1:**
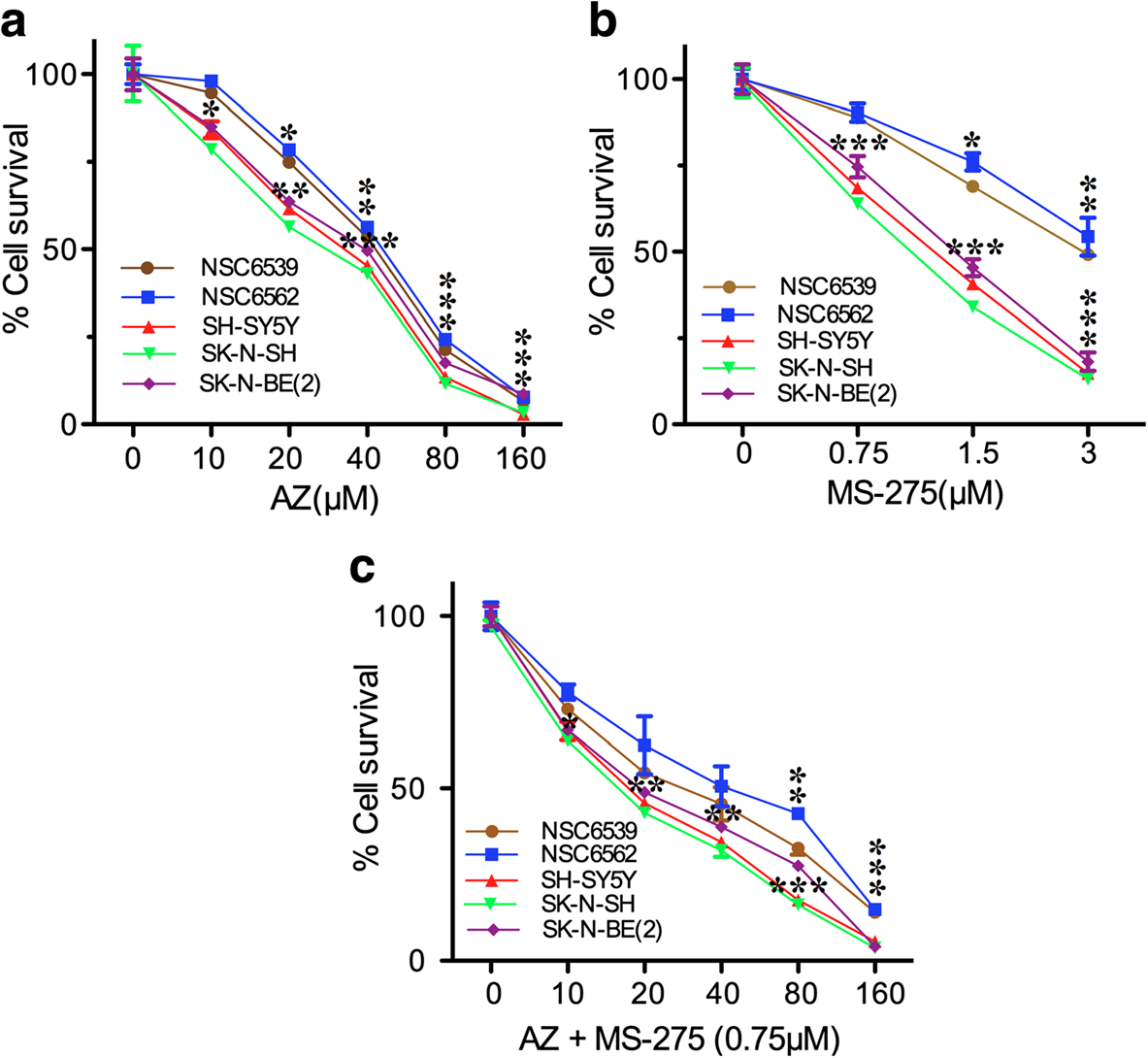
AZ and/or MS-275 treatments produced a dose-dependent reduction in NB cells. **a**-**c** present graphic and tabulated evidence for the dose response. Doses were AZ(0–160 μM), MS-275(0–3 μM) and AZ + MS-275(0–160 μM + 0.75 μM) for 48 h treatment of NSC6539, NSC6562, SH-SY5Y, SK-N-SH and SK-N-BE(2) cells compared to the untreated group. The greatest decrease in IC50 was observed for AZ + MS-275 combination as follow: SH-SY5Y = 17.5 μM, SK-N-SH = 16.5 μM and SK-N-BE(2) = 19.2 μM

Figure 1a-c shows that both AZ and MS-275 had a more moderate concentration-dependent inhibitory effect on neuronal stem cells while the effects of MS-275 and the AZ + MS-275 combination were significantly enhanced on all three tumor lines. The reduction in cell viability and IC50 values of NSC6539, NSC6562, SH-SY5Y, SK-N-SH and SK-N-BE(2) by AZ, MS-275, and AZ + MS-275 (48 h) shows the highest percent reduction with AZ + MS-275 in achievable plasma concentration (Tables 1 and 2). A significant difference in IC50 values for SH-SY5Y, SK-N-SH and SK-N-BE(2), indicates the potentiation of MS-275 effect by AZ. Interestingly, CDI analysis for the combination of AZ and MS-275 on different cell lines reveal that the combination is antagonistic at all concentrations on NSC6539 cells (CDI = 1.14–1.42) and additive on NSC6562 cells at concentrations above 20 μM AZ (CDI = 1). On NB cell lines (SH-SY5Y, SK-N-SH and SK-N-BE(2)) the combination was found to be additive at 40 μM and 80 μM of AZ (CDI = 1) and synergistic at 160 μM of AZ (CDI < 1). Since SH-SY5Y showed moderate resistance to AZ and/or MS-275, average concentration-response and IC50 compared to the other two NB cell lines, we chose to focus on SH-SY5Y cell line for the rest of the study. The AlamarBlue assay results also raised the question of effects on apoptosis and cell cycle as a measure of possible growth arrest and/or toxicity.

**Table 1:** Percentage of cell viability values

Cell line	Treatment modality	Concentration (μM)	Reduction in cell viability (%)	*p* value
NSC6539	AZ	10	6 ± 0.67	-
		20	26 ± 0.87	*p* < 0.05
		40	47 ± 0.07	*p* < 0.01
	MS-275	0.75	12 ± 0.85	-
		1.5	32 ± 0.97	*p* < 0.05
		3	51 ± 0.23	*p* < 0.01
	AZ + MS-275	10 + 0.75	28 ± 0.95	-
		20 + 0.75	46 ± 0.56	*p* < 0.05
		40 + 0.75	55 ± 0.50	*p* < 0.01
NSC6562	AZ	10	2 ± 0.07	-
		20	22 ± 0.47	*p* < 0.05
		40	45 ± 0.67	*p* < 0.01
	MS-275	0.75	10 ± 0.34	-
		1.5	24 ± 0.08	*p* < 0.05
		3	46 ± 0.39	*p* < 0.01
	AZ + MS-275	10 + 0.75	23 ± 0.86	-
		20 + 0.75	38 ± 0.50	*p* < 0.05
		40 + 0.75	50 ± 0.61	*p* < 0.01
SH-SY5Y	AZ	10	16 ± 0.20	*p* < 0.05
		20	39 ± 0.64	*p* < 0.01
		40	35 ± 0.58	*p* < 0.001
	MS-275	0.75	32 ± 0.55	*p* < 0.001
		1.5	60 ± 0.69	*p* < 0.001
		3	86 ± 0.78	*p* < 0.001
	AZ + MS-275	10 + 0.75	35 ± 0.85	*p* < 0.01
		20 + 0.75	55 ± 0.86	*p* < 0.001
		40 + 0.75	64 ± 0.78	*p* < 0.001
SK-N-SH	AZ	10	22 ± 0.59	*p* < 0.01
		20	34 ± 0.42	*p* < 0.01
		40	61 ± 0.03	*p* < 0.001
	MS-275	0.75	37 ± 0.99	*p* < 0.001
		1.5	66 ± 0.03	*p* < 0.001
		3	87 ± 0.19	*p* < 0.001
	AZ + MS-275	10 + 0.75	37 ± 0.85	*p* < 0.01
		20 + 0.75	58 ± 0.86	*p* < 0.001
		40 + 0.75	69 ± 0.95	*p* < 0.001
SK-N-BE(2)	AZ	10	15 ± 0.01	*p* < 0.01
		20	37 ± 0.61	*p* < 0.01
		40	61 ± 0.58	*p* < 0.001
	MS-275	0.75	26 ± 0.69	*p* < 0.001
		1.5	55 ± 0.38	*p* < 0.001
		3	82 ± 0.21	*p* < 0.001
	AZ + MS-275	10 + 0.75	34 ± 0.88	*p* < 0.01
		20 + 0.75	52 ± 0.88	*p* < 0.001
		40 + 0.75	62 ± 0.86	*p* < 0.001

Table 1 shows percentage of cell viability values of NSC6539, NSC6562, SH-SY5Y, SK-N-SH and SK-N-BE(2) by AZ(0–160 μM), MS-275(0–3 μM), and AZ + MS-275 (0–160 μM + 0.75 μM) treatments (48 h)

**Table 2:** Percentage of IC50 values

Cell	IC50 (μM)		
	AZ	MS-275	AZ + MS-275
NSC6539	53	2.8	23.5
NSC6562	56	3	35
SH-SY5Y	45	1.23	17.5
SK-N-SH	42	1	16.5
SK-N-BE(2)	49	1.48	19.2

Table 2 shows percentage of NSC6539, NSC6562, SH-SY5Y, SK-N-SH and SK-N-BE(2) by AZ (0–160 μM), MS-275 (0–3 μM), and AZ + MS-275 (0–160 μM + 0.75 μM) treatments (48 h)

**Fig. 2:**
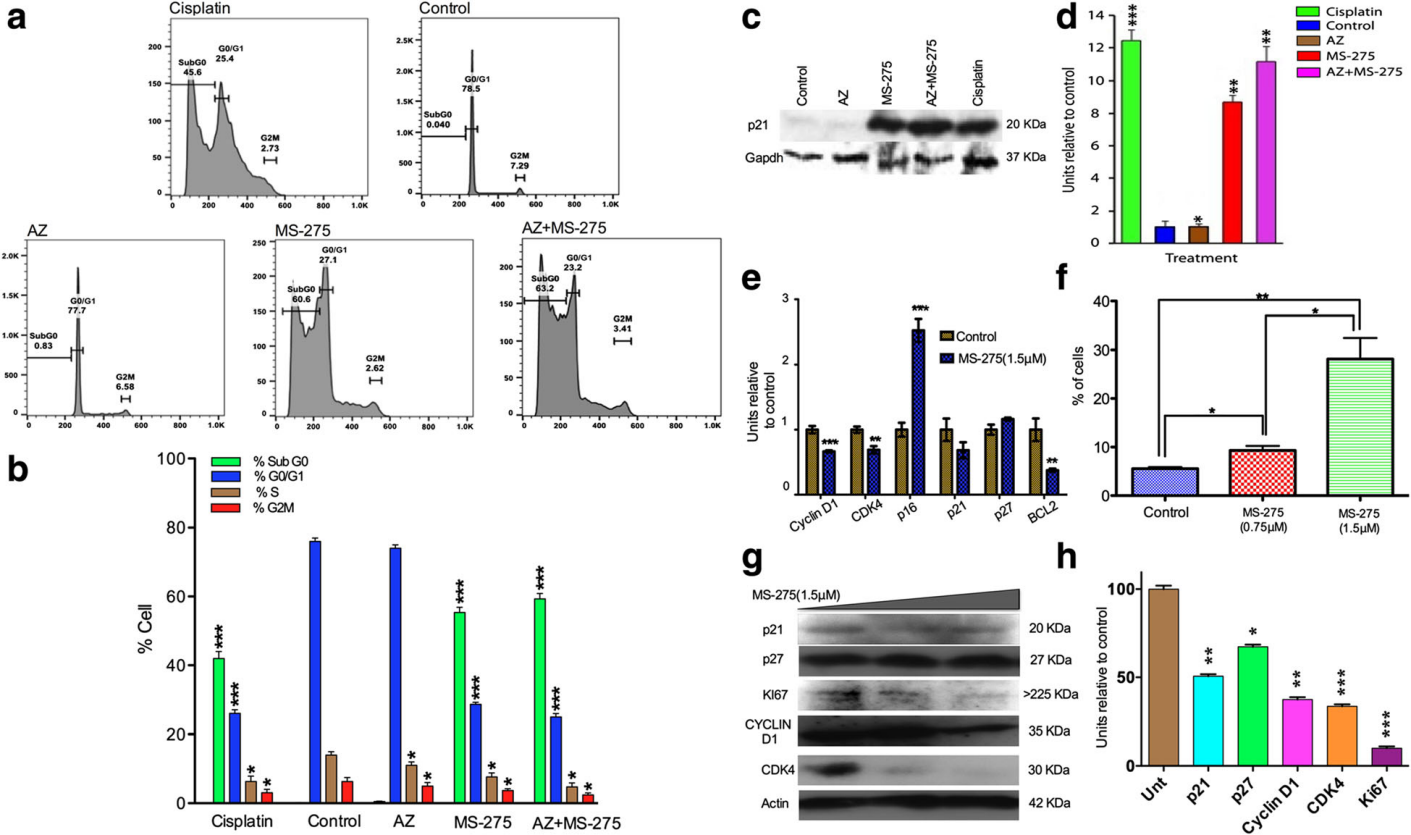
AZ and/or MS-275 treatments increased cell cycle arrest and apoptosis of NB cells. **a**-**b** show propidium iodide analysis of cell cycle at 48 h after treatment with AZ(40 μM), MS-275(1.5 μM) and AZ + MS-275 (40 μM + 0.75 μM) in NB SH-SY5Y, indicating increase entry of SH-SY5Y cells into SubG0-phase (0.6%, %57 and %61; *p* ≤ 0.001) with decrease into S-phase (13%, 9% and 4%; *p* ≤ 0.05) and G2/M-phase (6%, 2% and 3%; *p* ≤ 0.05). **c**-**d** show western blot analysis of cell cycle inhibitor, p21, indicates 48 h treatment of MS-275 and AZ + MS-275 treatments cause of 4.5 and 5.5 induction of p21 (*p* < 0.01) of SH-SY5Y cells compare to control, respectively. **e**-**f** show in-cell western (0.75 μM and 1.5 μM MS-275 treatment; 48 h) analyses of proteins associated with cell cycle arrest and apoptosis in NB SH-SY5Y cells. Results indicate that levels of cyclin D1 (0.666 ± 0.010%; *p* = 0.0006), CDK4 (0.690 ± 0.033%; *p* = 0.0002) and BCL2 decreased significantly (0.376 ± 0.014%; *p* = 0.0035) while p16 CDK inhibitor significantly increased (2.528 ± 0.101%; *p* = 0.0002). **g**-**h** show western blot data using lysates from cells treated 48 h with 0.75 μM and 1.5 μM MS-275. p21 and p27 showed constant levels following treatment whereas cyclin D1 and CDK4 were reduced in expression. Expression of Ki67 proliferative marker decreased as well

We characterized the effect on apoptosis and cell cycle using a propidium iodide (PI) based FACS analysis. Using a dose less than the IC50 dose, AZ (40 μM vs. 45 μM), MS-275(1.5 μM vs. 2.36 μM) and AZ + MS-275(40 μM + 0.75 μM) it was found that AZ, MS-275 and AZ + MS-275 treatments increase entry of SH-SY5Y cells into SubG0-phase (0.6%, %57 and %61) with decrease into S-phase (13%, 9% and 4%) and G2/M-phase (6%, 2% and 3%), significantly following a 48 h dosage of AZ(40 μM), MS-275(1.5 μM) and AZ + MS-275(40 μM +0.75 μM), respectively (Fig. 2a-b). In addition, western blot analysis of cell cycle inhibitor, p21, shows that MS-275 and AZ + MS-275 treatments (48 h) cause of 4.5 and 5.5 induction of p21 (*p* < 0.01) of SH-SY5Y cells compare to control, respectively (Fig. 2c-d). Results would suggest that AZ, MS-275 and AZ + MS-275 treatments are inducing apoptosis and preventing entry into S and G2/M-phases. This was further characterized by in-cell western and western blot analysis of cell cycle checkpoints, demonstrating a 2.5 fold induction of p16 (85% ± 0.19%; *p* < 0.001), a 1.97 fold induction of p21 (50% ± 0.64%; *p* < 0.001), a 1.48 fold induction of p27 (34% ± 0.31%; *p* < 0.05), cyclin dependent kinase inhibitors (Cdki), and downregulation of CDK4 (33% ± 0.15%; *p* < 0.01) and cyclin D1 (31% ± 0.35%; *p* < 0.001) following a 48 h dosage of 1.5 μM MS-275, respectively (Fig. 2e-f). These results were confirmed by western blot analysis (Fig. 2 g-h). Furthermore, western blot analysis of the expression of the Ki67 proliferative marker showed a down regulation of Ki67 (Fig. 2 g-h). Ki67 is expressed at all points during the cell cycle, except in G0. It should be noted that inductions of Cdki p21 occurred at the lower concentration of MS-275 but decreased somewhat at the higher concentrations suggesting a more complex epigenetic regulatory mechanism. Overall Cdki inductions varied except for p16. We also conducted a PI cell cycle analysis to determine the percentage of sub-G1 cell.

**Fig. 3:**
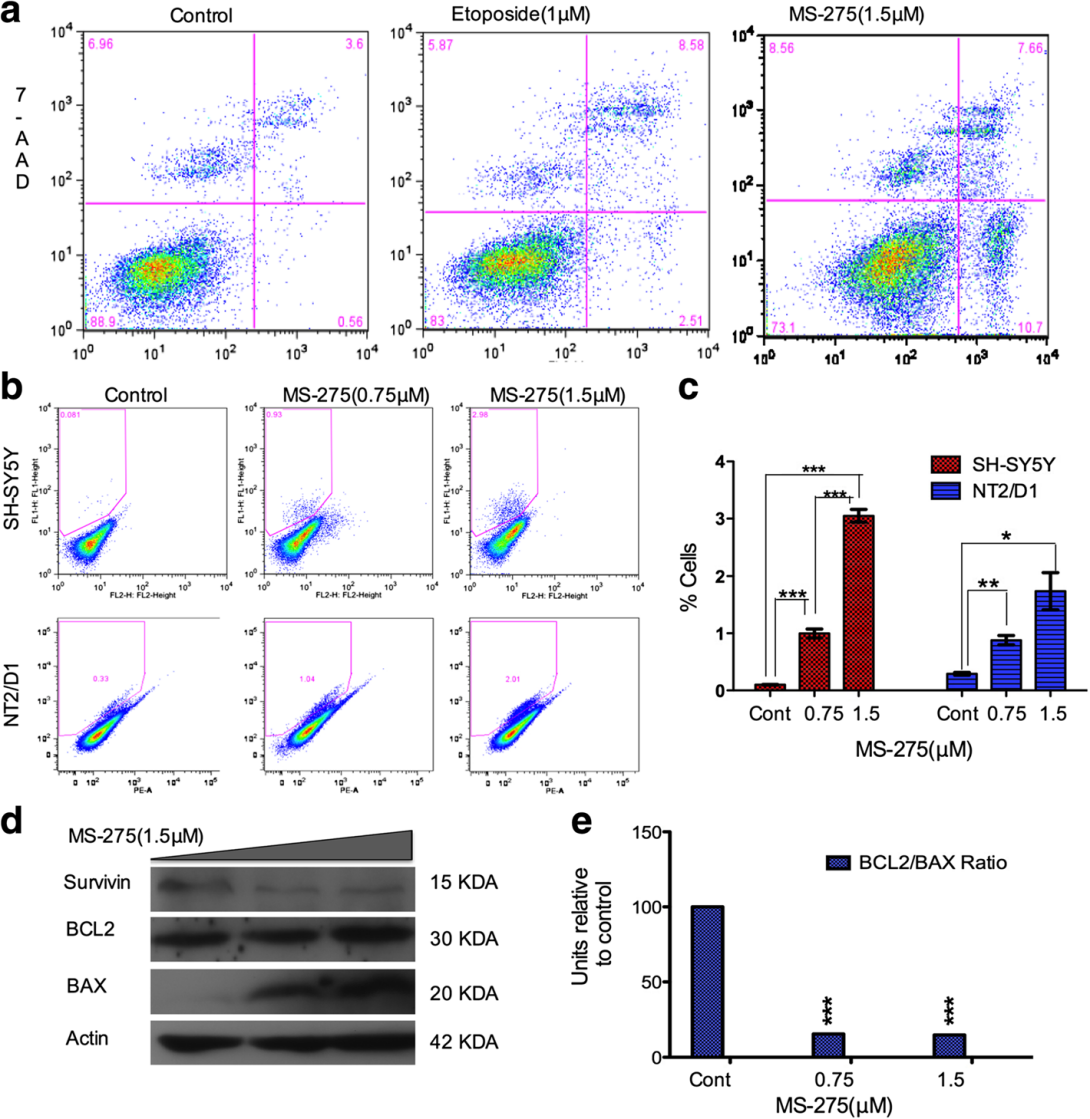
**a**-**b** show the results of FACs analysis for 7-AAD and Annexin staining. Results demonstrate that compared to etoposide, MS-275 induces entry into early and late stage of apoptosis in 10.7% versus 2.51% and 7.66% versus 8.58% of cells, respectively. **c** show cleaved caspase 3 expression after 48 h treatment with 0.75 μM and 1.5 μM MS-275 that yielded 1% and 3% expression, respectively (*p* = 0.0003 and *p* < 0.0001, respectively as compared to control and *p* = 0.0001 when comparing doses). **d**-**e** show increased expression of apoptotic protein BAX and decreased BCL2/BAX ratio with coordinate decrease in survival following 0.75 μM and 1.5 μM MS-275 treatment to 15.59% and 14.86% of control. In addition, survivin expression reduced following 0.75 μM and 1.5 μM MS-275 treatment to 7.8% and 11%, respectively

Since cell death due to mechanical damage is also accounted for in sub-G1 cell analysis, we also assessed Annexin/7-AAD and cleaved-caspase 3 expressions by FACs. Annexin stains cells in the early stage of apoptosis, while positive stain for 7-AAD indicates loss of membrane integrity. Cells at end stage of apoptosis are characterized by double staining for Annexin and 7-AAD. The results of FACs analysis demonstrated that MS-275 induces both early (10.7%) and late stage apoptosis (7.66%). The parallel assay with a clinically used chemotherapeutic 1 μM Etoposide (the IC50 dose) showed 2.51% and 8.58% induction of early and late stage apoptosis, respectively (Fig. 3a and Table 3). In addition, using the apoptotic indicator, cleaved caspase 3, 0.75 μM and 1.5 μM MS-275 after 48 h treatment yielded significantly 1% and 3% expression of cleaved-caspase 3, respectively (*p* = 0.0003 and *p* < 0.0001 respectively as compared to control and *p* = 0.0001 when comparing doses) (Fig. 3b-c). The pro-apoptotic effect of MS-275 was further investigated by western blot analysis of BCL2 and BAX expression. The results demonstrated that BCL2/BAX ratio is reduced (19.6% of control) by 1.5 μM MS-275 treatment (48 h);(Fig. 3d), indicating a potent apoptotic effect. MS-275 increases the expression of apoptotic protein BAX, hence decreasing the BCL2/BAX ratio, and coordinate with decrease in survival. Western blot analysis also demonstrated that survivin, a potent inhibitor of apoptosis, is reduced in expression following MS-275 treatment (Fig. 3e). Here NT2/D1 is a neuronal subclone of the teratomas NT2 cell line used in comparison, and as it is more stem cell like.

**Table 3:** Percentage of apoptosis values

	Unstained (Lower left)	Annexin (Lower right)	7-AAD (Upper left)	Annexin 7-AAD (Upper right)
Control	90	0.6	6.80	3.9
Etoposide (1 μM)	85	2.57	5.90	8.68
MS-275 (1.5 μM)	72.18	10.11	8.60	7.06

Table 3 shows the parallel assay with a clinically used chemotherapeutic Etoposide (1 μM);(2.51% and 8.58%) and MS-275 (1.5 μM);(10.7% and 7.66%) showed induction of early and late stage apoptosis, respectively

### AZ, MS-275 and AZ + MS-275 treatments reduced migration capacity in NB SH-SY5Y cells

**Fig. 4:**
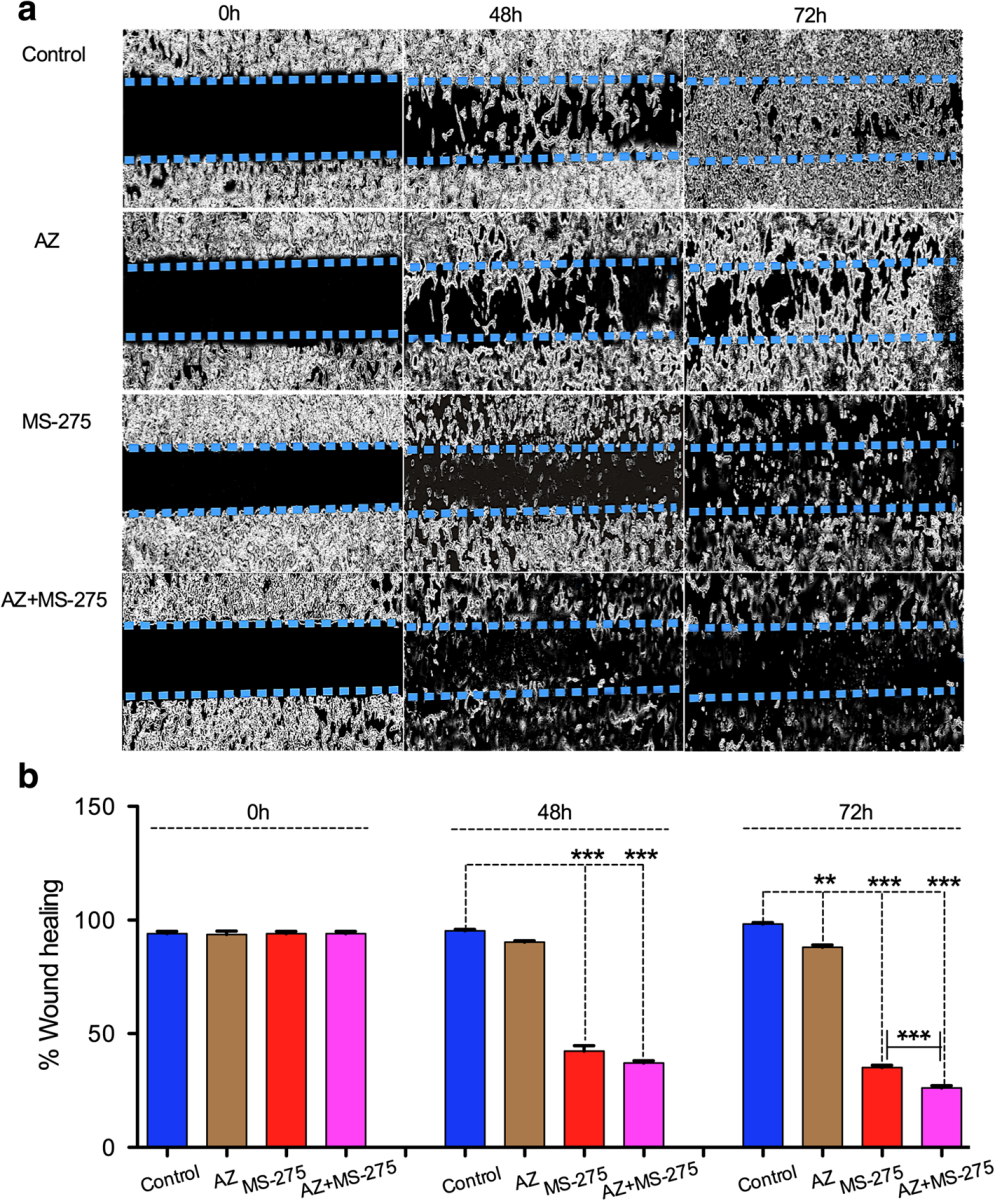
AZ and/or MS-275 treatments decreased migration capacity of NB cells. **a**-**b** represent the wound healing assay for AZ (40 μM), MS-275 (1.5 μM) and AZ + MS-275 (40 μM + 0.75 μM) treatment compared to untreated group in SH-SY5Y cells. AZ caused a 10 ± 0.35% (*p* = 0.025, 48 h) and 12 ± 0.85% (*p* < 0.01,72 h) inhibition while MS-275 caused a 42 ± 0.13% (*p* < 0.001, 48 h) and 65 ± 0.30% (*p* < 0.001, 72 h) inhibition in migration. The AZ + MS-275 combination significantly inhibited migration with a 63 ± 0.37% (*p* < 0.001, 48 h) and 74 ± 0.25% (*p* < 0.001, 72 h) inhibition in migration ability

Previous studies had shown that both AZ and MS-275 reduce migration capacity in bladder and liver cancer cells [27, 28]. In the current study, we show the reduction of migration capacity in AZ (40 μM), MS-275 (1.5 μM) and AZ + MS-275 (40 μM + 0.75 μM) treated NB SH-SY5Y cells in Table 4. In Fig. 4a it is evident that AZ alone has an obvious inhibitory effect, and greatly enhanced by MS-275 at 48 h. The difference between MS-275 alone and the combo treatment was statistically significant (*p* < 0.001) at both 48 h and 72 h. AZ therefore enhances the inhibitory effect of MS-275 on SH-SY5Y cell migration capacity (Fig. 4a-b). In this assay the cytotoxicity of the agents is demonstrated overtly because of the initial cell confluence and may predict subsequent in vivo effects.

**Table 4:** Percentage of cell migration capacity values

Time	Control	48 h	72 h
Treatment	% Cell migration capacity		
Untreated	94	95.33	96.98
AZ	93.66	90.3	85.87
	-	-	*p* < 0.01
MS-275	94	42.33	38.56
		*p* < 0.001	*p* < 0.001
AZ + MS-275	95	37	26
		*p* < 0.001	*p* < 0.001

Table 4 shows percentage of cell migration capacity values of SH-SY5Y by AZ (40 μM), MS-275 (0.75 μM) and AZ + MS-275 (40 μM + 0.75 μM) treatments (48 h and 72 h)

### AZ significantly potentiates the inhibitory effect of MS-275 on tumorigenesis in NB SH-SY5Y xenografts

**Fig. 5:**
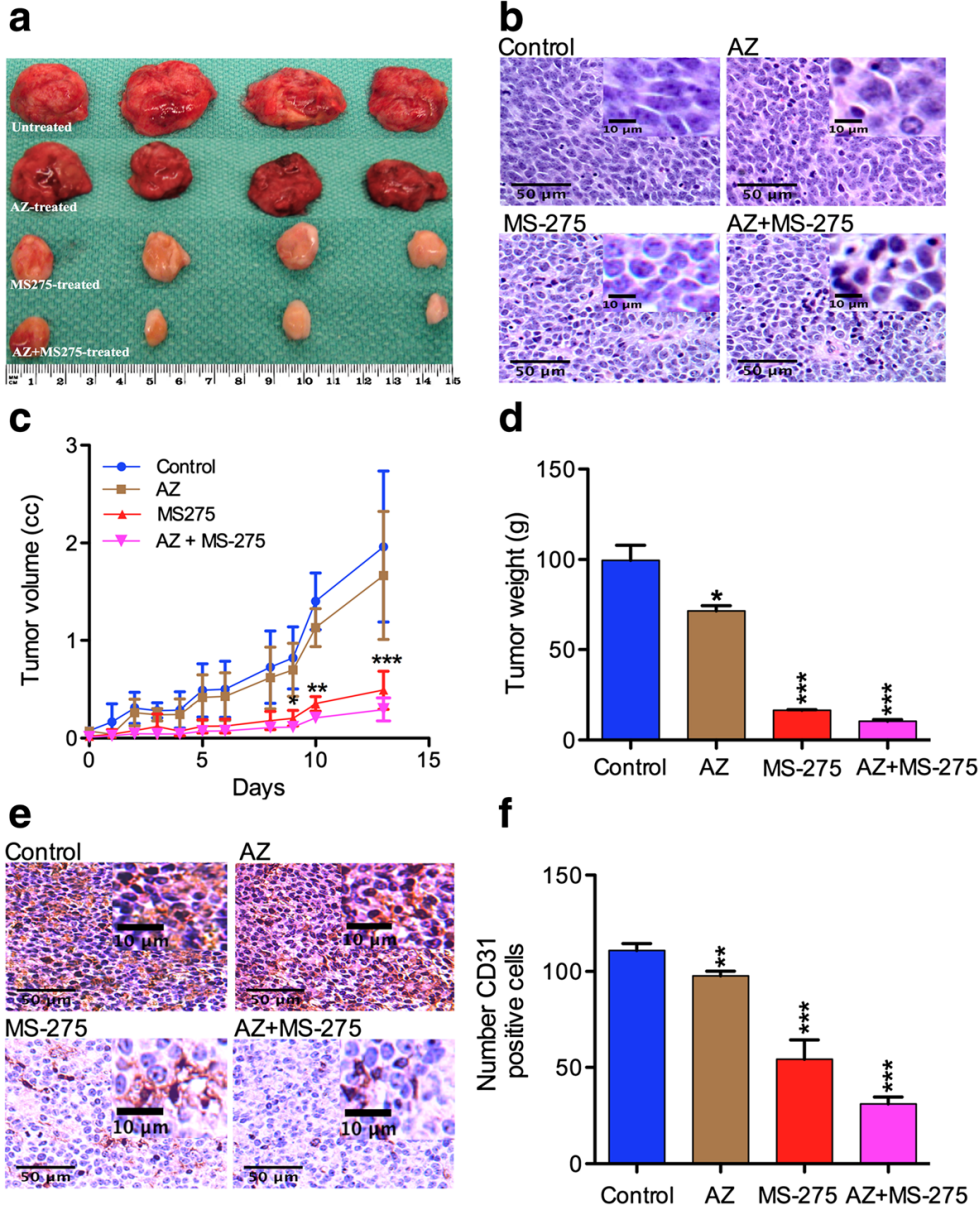
AZ and/or MS-275 inhibited tumor growth of NB xenografts. **a** presents the gross morphology, **b** H&E histology, **c** volume, **d** weight, **e** CD31 staining and **f** the percentage of CD31 positive cells after 14 days treatment with AZ and/or MS-275 compared to the untreated group in SH-SY5Y xenografts. AZ caused 13% ± 0.29%, MS-275 43% ± 0.50% and AZ + MS-275 60% ± 0.48% (*p* = 0.0009) inhibition in tumor growth. Tumor weights were reduced by 29 ± 0.2% (*p* = 0.193) for AZ, 83% ± 0.74% (*p* < 0.001) for MS-275 and 89.5 ± 0.49% (*p* < 0.001) for AZ + MS275. AZ caused a 13% ± 0.98%(*p* = 0.006), MS-275 a 52% ± 0.0.94% (*p* = 0.0008) and AZ + MS-275 a 73% ± 0.92% (*p* < 0.0001) decrease in the number of CD31 positive cells compared to the control

A concentration of 24.5 mg/kg MS-275 reduced growth of NB KCNR cell line as orthotopic xenografts [8]. A 14-day treatment protocol with 40 mg/kg AZ and/or 20 mg/kg MS-275 was devised based on the observed IC50 results and previously used safe doses in other preclinical studies [8, 29]. The inhibition of tumor volume is presented in Table 5. Figure 5a grossly shows a dramatic reduction in tumor growth and volume after MS-275 (20 mg/kg) and significantly greater when AZ (40 mg/kg) was added to the treatment. Figure 5b shows the changes in histology suggesting phenotypic alterations while Fig. 5c and d graphically reflect large reductions of volumes and weights, over the 14 days treatment period. The extirpated tumors also revealed grossly that both MS-275 and AZ + MS-275 markedly reduced the hematogeneous appearance of the tumors suggesting significant loss of vascularization (Fig. 5a-b). The significant reductions in tumor growth and weight were greatest with AZ + MS-275 (Table 5). The significant anti-tumor growth potentiation effect of AZ on MS-275 suggested an additive effect for this combination. IHC results revealed a possible explanation for the dramatic reduction in tumor volumes in that there was a strong inhibition of angiogenesis as revealed with staining for the angiogenesis marker (CD31) (Fig. 5e). Compared to the untreated group, expression of the CD31 was most significantly reduced with the combination AZ + SFN (Table 5; Fig. 5f).

**Table 5:** Percentage of tumor volume, weight and expression of CD31 values

Treatment modality	Concentration (mg/kg)	% Inhibition of tumor volume (*p* value)	% Reduction in tumor weight (*p* value)	% Expression of CD31 marker (*p* value)
AZ	40	13 ± 0.29 (*p* = 0.009)	29 ± 0.2 (*p* = 0.193)	87 ± 0.98 (*p* = 0.006)
MS-275	20	43 ± 0.50 (*p* = 0.001)	83 ± 0.74 (*p* < 0.001)	48 ± 0.94 (*p* = 0.008)
AZ + MS-275	40 + 20	60 ± 0.48 (*p* = 0.001)	89.5 ± 0.94 (*p* < 0.001)	27 ± 0.92 (*p* < 0.001)

Table 5 shows percentage of tumor volume and weight reduction and expression of CD31 values of SH-SY5Y tumors by AZ (40 mg/kg), MS-275 (20 mg/kg) and AZ + MS-275 (40 + 20 mg/kg) treatments (14D)

### AZ, MS-275 and the AZ + MS-275 treatments induce apoptosis in NB SH-SY5Y xenograft cells

**Fig. 6:**
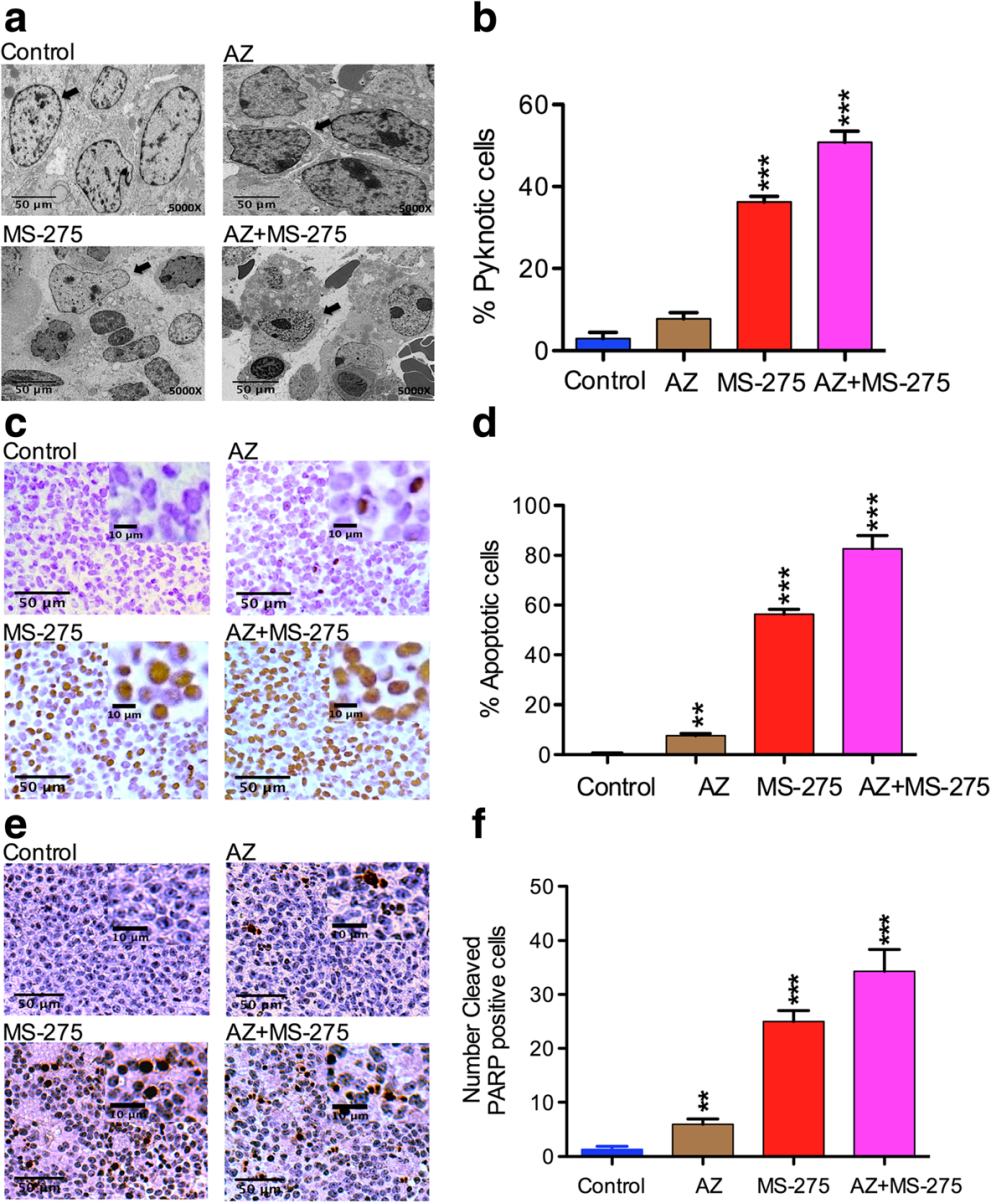
AZ and/or MS-275 treatments increase the apoptotic TUNEL index in NB xenografts. **a**-**b** present ultrastructural details [x5000, cytoplasmic (arrow)] including the percentage of pyknotic cells. **c**-**d** present results from the TUNEL assay (x20 and x40) for detection of apoptotic cells (*arrow*) and the number of apoptotic positive cells after 14 days treatment with AZ, MS-275 and AZ + MS-275 compared to untreated group in SH-SY5Y xenografts. The number of TUNEL positive cells increased modestly after treatment with AZ (7 ± 0.66%; *p* = 0.0015), moderately after MS-275 (56 ± 0.33%; *p* < 0.001) and significantly after AZ + MS-275 (82 ± 76%; *p* < 0.001) compared to the untreated group. **e**-**f** present the expression of the apoptotic marker (cleaved PARP) that was significantly induced by AZ (12% ± 0.16%; *p* = 0.001), MS-275 (63% ± 0.33%; *p* = 0.0001) and further by AZ + MS-275 (78% ± 0.09%; *p* = 0.0001)

The initial histopathological assessment of the residual tumors (Fig. 5a-e) revealed reductions in cell density and size, presence of pyknotic nuclei and reduced nuclear size, most prominently in the case of AZ + MS-275. This suggested that apoptosis could account for cell loss. To further confirm the histological changes, we performed electron microscopy on the tumor xenografts. Ultrastructural analysis revealed cells with degradative cytoplasmic changes [8, 30, 31] and nuclear fragmentation (pyknotic cells) indicative of apoptosis in SH-SY5Y xenografts. AZ + MS-275 treated cells had the highest number of pyknotic cells (Table 6; Fig. 6a-b), further supported the histological finding that AZ potentiated the apoptotic effect of MS-275. Next, we assessed apoptosis with the TUNEL assay on the xenografts (Table 6). We found that AZ + MS-275 treated cells had the highest percent TUNEL positive cells (Fig. 6c-d), and significantly greater than MS-275 alone. To further confirm the apoptotic process, we performed IHC to study the effect of AZ, MS-275 and AZ + MS-275 treatments on expression of the apoptotic marker (cleaved PARP) in the SH-SY5Y xenografts. Compared to the untreated group, expression of the apoptotic marker (cleaved PARP) was significantly induced, versus untreated controls (Table 6; Fig. 6e-f). Thus, AZ showed an overt potentiation of the anti-tumor effect of MS-275 in the SH-SY5Y xenografts.

**Table 6:** Percentage of pyknotic, TUNEL and Cleaved PARP positive cells values

Treatment modality	% Pyknotic cells (*p* value)	% TUNEL positive cells (*p* value)	% Cleaved PARP positive cells (*p* value)
AZ	8 ± 0.6 (*p* < 0.001)	7 ± 0.66 (*p* = 0.0015)	12 ± 0.7 (*p* < 0.01)
MS-275	36 ± 0.45	56 ± 0.33 (*p* < 0.001)	50 ± 0.23 (*p* < 0.001)
AZ + MS-275	51 ± 0.83 (*p* < 0.001)	82 ± 0.76 (*p* < 0.001)	69 ± 0.35 (*p* < 0.001)

Table 6 shows the percentage of pyknotic, TUNEL and Cleaved PARP positive cells values of SH-SY5Y tumors by AZ (40 mg/kg), MS-275 (20 mg/kg) and AZ + MS-275 (40 + 20 mg/kg) treatments (14D)

### AZ, MS-275 and AZ + MS-275 reduce expression of mitotic and proliferative markers in NB SH-SY5Y xenografts

**Fig. 7:**
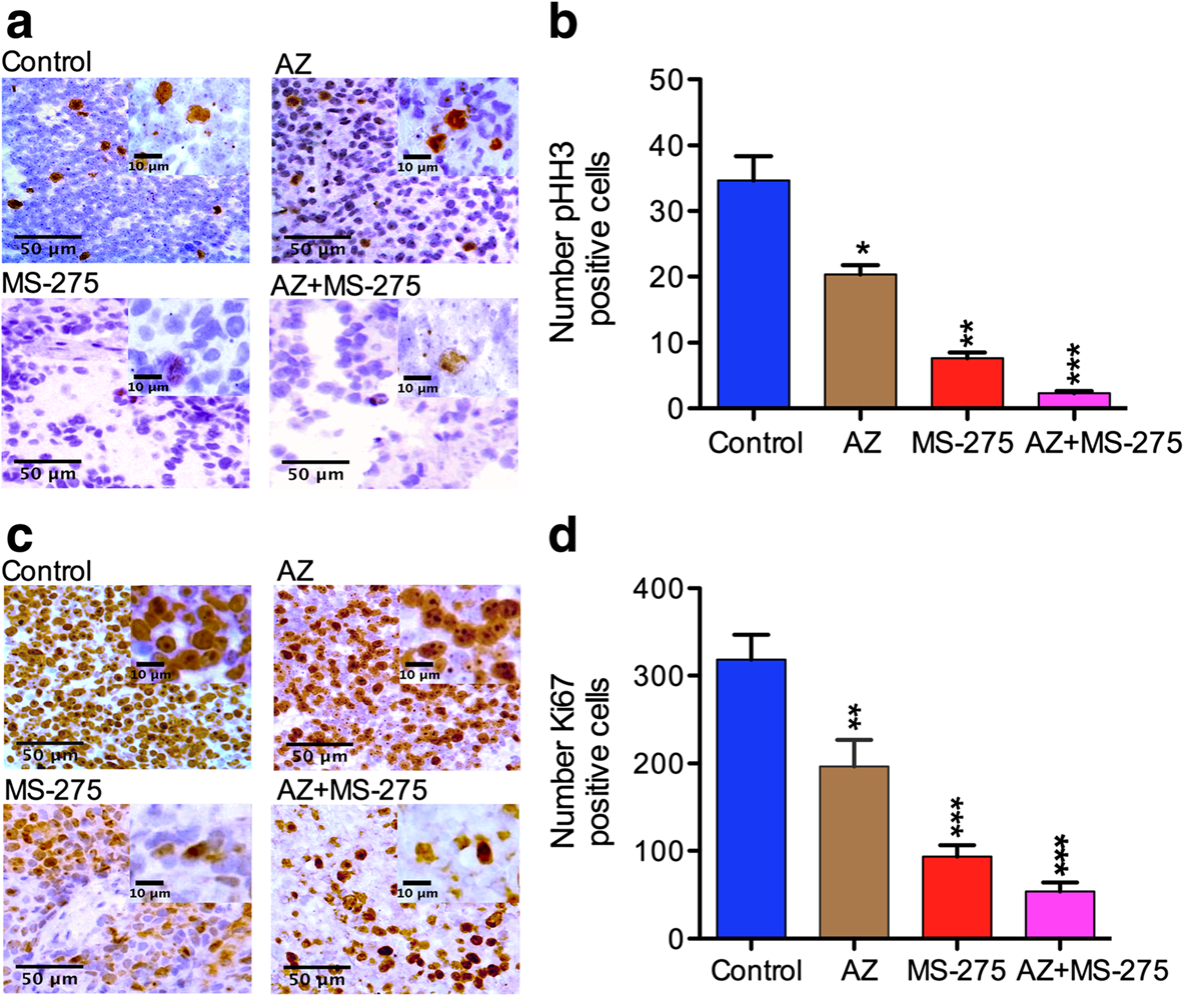
AZ and/or MS-275 treatments affected the mitosis and proliferation of NB xenografts. **a**-**b** present the IHC study (x20 and x40) of the mitotic index (pHH3, *arrow*) and the number of pHH3 positive cells. **c**-**d** present the immunodetection and number of Ki67 positive cells (*arrow*) after 14 days treatment with AZ, MS-275 and AZ + MS-275 compared to untreated group in SH-SY5Y xenografts. Data shows that pHH3 expression was significantly reduced by AZ (42 ± 0.43%; *p* = 0.02), MS-275 (78 ± 0.16%; *p* = 0.002) and further by AZ + MS-275 (94 ± 0.05%; *p* = 0.001). Ki67 expression was moderately reduced by AZ (38 ± 0.70%; (*p* = 0.007), strongly reduced by MS-275 (70 ± 0.25%; *p* = 0.0002) and further reduced by AZ + MS-275 (84 ± 0.73%; *p* = 0.0001)

The results from the apoptosis assessment of treated xenografts suggested strong effects on tumor cell proliferation. We therefore performed IHC to study the effect of AZ, MS-275 and AZ + MS-275 treatments on expression of mitotic (phosphohistone-H3; pHH3) and proliferation (Ki-67) markers in the SH-SY5Y xenografts. Compared to the untreated group, expression of the mitotic marker (pHH3) was moderately reduced after AZ treatment alone, significantly after MS-275 treatment and further enhanced with the combination AZ + MS-275 (Table 7; Fig. 7a-b). In a similar manner expression of the proliferative marker, Ki67 was significantly reduced by all treatments and the most by AZ + MS-275 (Table 7; Fig. 7c-d). Thus, the large reductions in tumor growth produced by MS-275 and the AZ + MS-275 combination are additionally reflected in significant reductions in mitosis and proliferation paralleled by increased apoptosis. It is noteworthy that the potent inhibitory effect of AZ alone was revealed using these markers, and potentiation of MS-275 was further confirmed.

**Table 7:** Percentage of pHH3 and Ki67 positive cells values

Treatment modality	% Expression of pHH3 positive cells (*p* value)	% Expression of Ki67 positive cells (*p* value)
AZ	58 ± 0.66 (*p* < 0.05)	61 ± 0.77 (*p* = 0.01)
MS-275	22 ± 0.11 (*p* < 0.01)	29 ± 0.42 (*p* < 0.001)
AZ + MS-275	6 ± 0.73 (*p* < 0.001)	16 ± 0.85 (*p* < 0.001)

Table 7 shows percentage of pHH3 and Ki67 positive cells values of SH-SY5Y tumors by AZ (40 mg/kg), MS-275 (20 mg/kg) and AZ + MS-275 (40 + 20 mg/kg) treatments (14D)

### AZ and/or MS-275 treatment reduced expression of HIF1-α and CAIX in NB SH-SY5Y xenograft

**Fig. 8:**
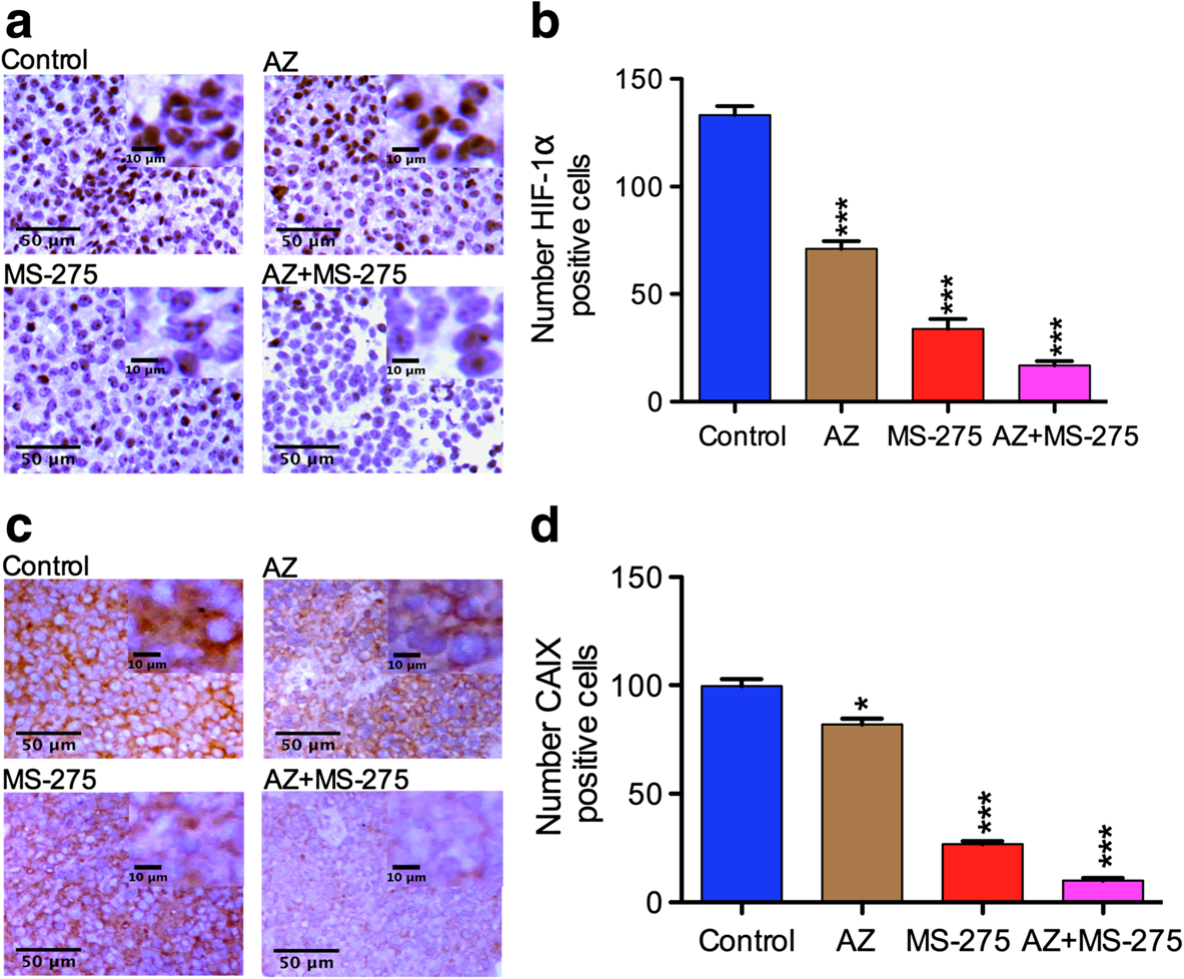
AZ and/or MS-275 treatments affected hypoxia response and CAIX in NB xenograft cells. **a**-**b** present the IHC study (x20 and x40) on HIF1-α expression, (*arrow*) and the number of HIF-1α positive cells. **c**-**d** present CAIX expression (*arrow*) and the number of CAIX positive cells after 14 days treatment with AZ and/or MS-275 compared to untreated group in SH-SY5Y xenografts. HIF-1α expression was significantly reduced by AZ (29 ± 0.7%; *p* < 0.001), MS-275 (66 ± 0.23%; *p* < 0.001) and further enhanced in AZ + MS-275 (83 ± 0.67%; *p* < 0.001). CAIX expression was significantly reduced by AZ (18 ± 0.11%; *p* = 0.0138), MS-275 (73 ± 0.33%; *p* < 0.001) and markedly by AZ + MS275 (90 ± 0.16%; *p* < 0.001)

Given the remarkable reductions in vascularization it might be surmised that the tumors would experience enhanced hypoxia under the treatments and thereby increased hypoxia induced gene expression. However, we performed IHC on the xenografts and did observe that control untreated tumors significantly expressed HIF1-α and its downstream target CAIX (Table 7; Fig. 8a-d). We further found that the number of HIF1-α positive cells decreased significantly after all treatments and markedly after AZ + MS-275 (Table 8; Fig. 8a and b). Additionally, a markedly enhanced reduction in CAIX expression (membrane localization) after AZ + MS-275 (Table 8; Fig. 8c and d) paralleled that of HIF1-α. The major reduction in CAIX staining after AZ + MS-275 may reflect much more than a loss of viable cells supporting the concept of AZ potentiation of the epigenetic alterations in expression.

**Table 8:** Percentage of HIF-1α and CAIX positive cells values of SH-SY5Y tumors

Treatment modality	% Expression of HIF-1α positive cells (*p* value)	% Expression of CAIX positive cells (*p* value)
AZ	71 ± 0.70 (*p* < 0.001)	82 ± 0.11 (*p* = 0.0138)
MS-275	34 ± 0.23 (*p* < 0.001)	27 ± 0.33 (*p* < 0.001)
AZ + MS-275	17 ± 0.73 (*p* < 0.001)	10 ± 0.16 (*p* < 0.001)

Table 8 shows the percentage of SH-SY5Y tumors by AZ (40 mg/kg), MS-275 (20 mg/kg) and AZ + MS-275 (40 + 20 mg/kg) treatments (14D)

### AZ + MS-275 treatment significantly reduces the in vivo tumorigenic potential of NB SH-SY5Y xenograft cells

**Fig. 9:**
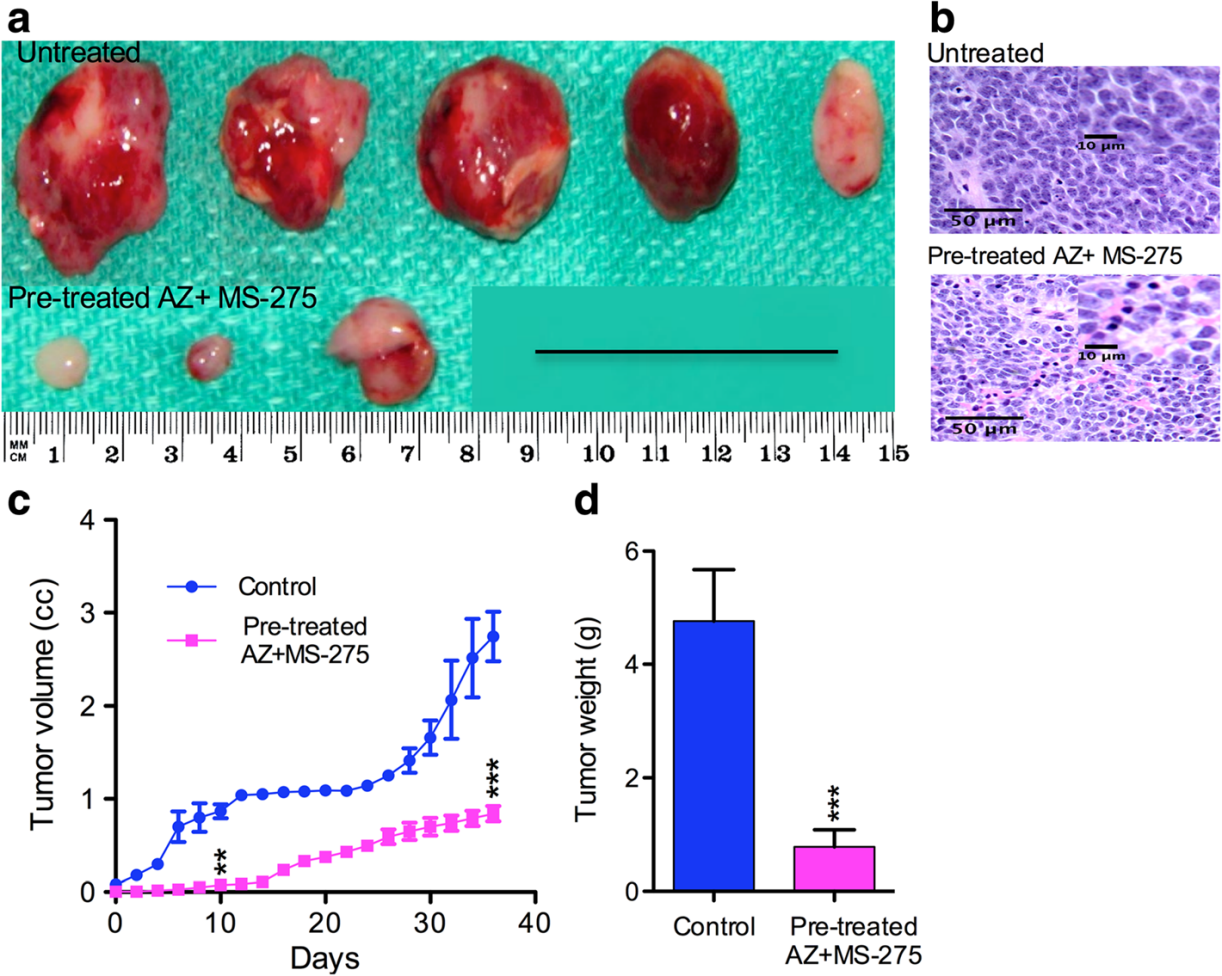
AZ + MS-275 decreased the in vivo tumorigenic potential of pretreated NB SH-SY5Y cells. **a** presents the in vivo serial heterotransplantation study (x20 and x40) with pretreated NB xenograft cells showing the morphology, **b** H&E histology, **c** volume and **d** weight after 37 days treatment with AZ + MS-275 compared to untreated group in SH-SY5Y xenografts. Notably, AZ + MS-275 caused a significant reduction in tumor volumes (69 ± 0.84%; *p* = 0.0002) and weights (81 ± 0.78%; *p* = 0.0001) after 37 days compared to xenografts generated from untreated cells

The ultimate test for loss of tumorigenic potential is whether treatments abrogate formation of tumors, accomplished by conducting serial heterotransplantation. To test this, after the initial round of treatments with the most effective AZ + MS-275 combination we serially heterotransplanted a second set of 3x10^4^ cells into NOD/SCID mice and in comparison to a similar number of untreated cells. We found that the combination of AZ + MS-275 caused a 69% ± 0.84% (*p* = 0.0002) inhibition in resulting tumor volumes and weights (81% ± 0.78%; *p* = 0.0001) respectively after 38 days (Fig. 9a-d). Note however that only 3/5 injections yielded any tumor mass. A marked phenotypic alteration in morphological appearance (Fig. 9b) resembled the treated tumors in Fig. 5a-e. These results suggest that the AZ + MS-275 combination might permanently alter cell phenotype and affect the presumptive CSCs in the tumor.

### AZ, MS-275 and AZ + MS-275 treatment affects stem cell marker expression in NB SH-SY5Y xenograft cells

**Fig. 10:**
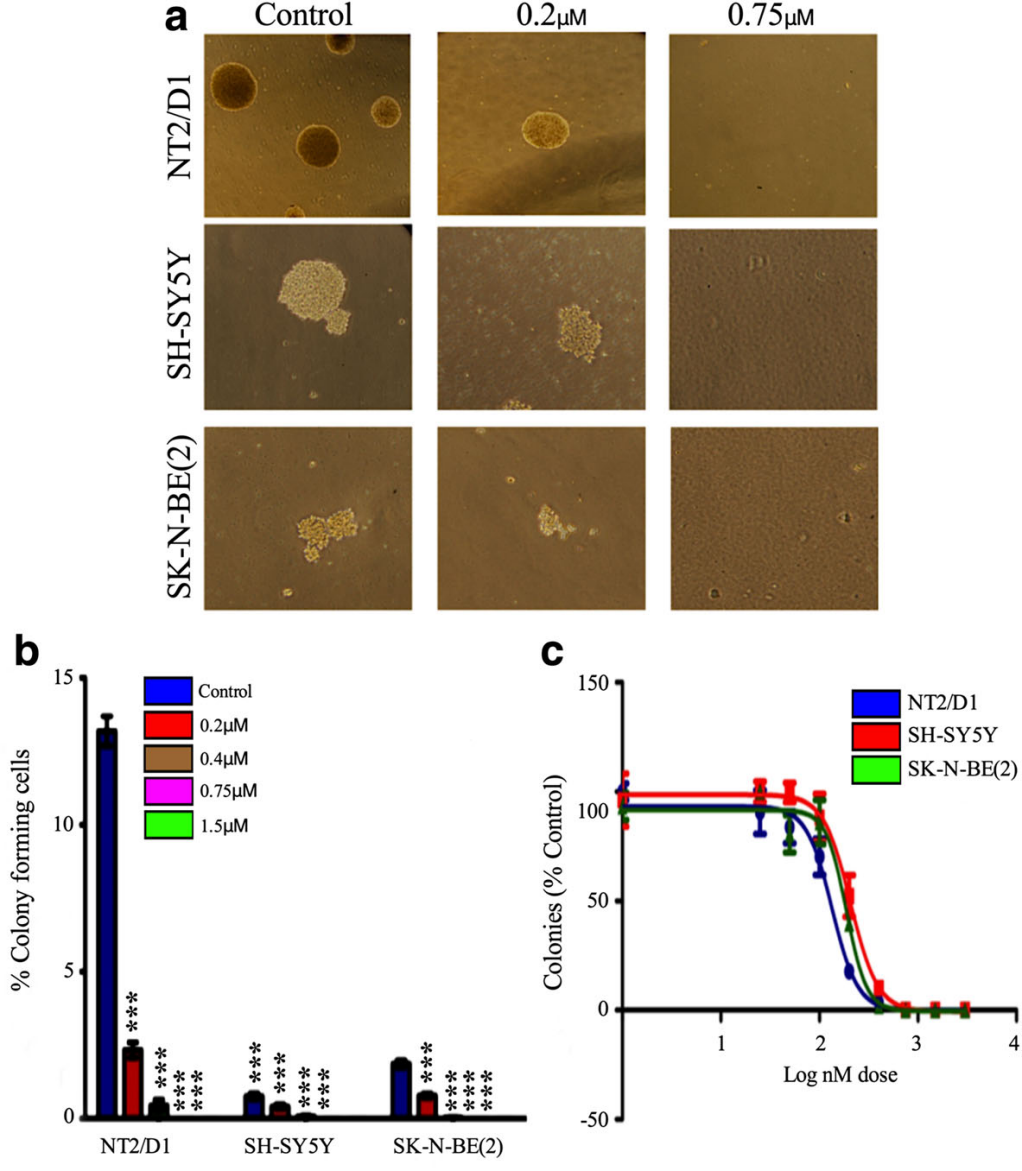
MS-275 treatment reduced clonogenic potential of NB and teratocarcinoma cells. **a** clonogenic potential was negatively affected with increasing doses of MS-275. **b** dose response curves of MS-275 treatment of NT2/D1, SH-SY5Y and SK-N-BE(2) over a 2 week growth period in methycellulose compared to control. Similarly, clonogenic capacity was negatively affected in all three cell lines despite different initial clonogenic efficiencies. IC50 values were calculated to be 0.13 μM, 0.20 μM and 0.18 μM for NT2/D1, SH-SY5Y and SK-N-BE(2), respectively. **c** representative images for control, 0.2 μM and 0.75 μM MS-275 treatments in methycellulose clonogenic assay run on NT2/D1 and SK-N-BE(2)

**Fig. 11:**
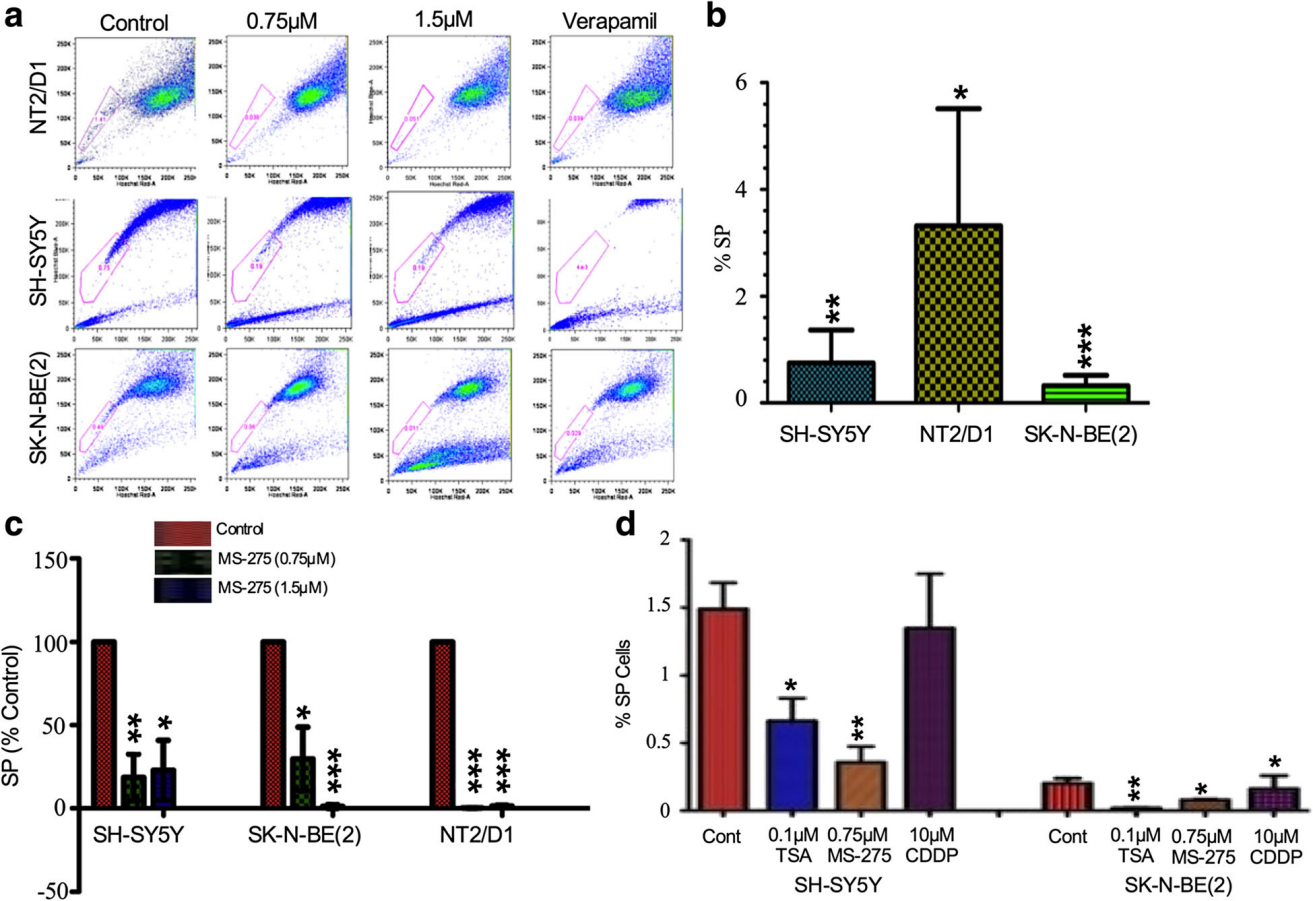
MS-275 treatment decreased the SP fraction of NB and teratocarcinoma cells. **a** using Hoechst 33342 dye exclusion, the SP fraction in NT2/D1, SH-SY5Y and SK-N-BE(2) was determined to be 3.32 ± 1.26%, 0.74 ± 0.35% and 0.32 ± 0.11%, respectively. **b** representative FACs SP profiles for SH-SY5Y control and cells treated with 1.5 μM MS-275, 100nM TSA and 10 μM CDDP. Gating was determined by verapamil negative control. **c** normalizing for control, 0.75 μM MS-275 reduced the SP fraction significantly (*p* < 0.0001, *p* = 0.0098 and *p* = 0.0237). Similar treatments with 1.5 μM MS-275 also reduced the SP population in NT2/D1, SH-SY5Y and SK-N-BE(2) significantly (*p* < 0.0001, *p* = 0.0177 and *p* < 0.0001). **d** low nM doses, 0.1 μM HDACi TSA and 0.75 μM MS-275, significantly reduced the SP fraction of both NB lines but greater in SK-N-BE(2), a MYCN amplified cell line (*p* = 0.0062, *p* = 0.045 respectively); in contrast highly toxic levels of CDDP did significantly alter the SP fraction (*p* > 0.05)

**Fig. 12:**
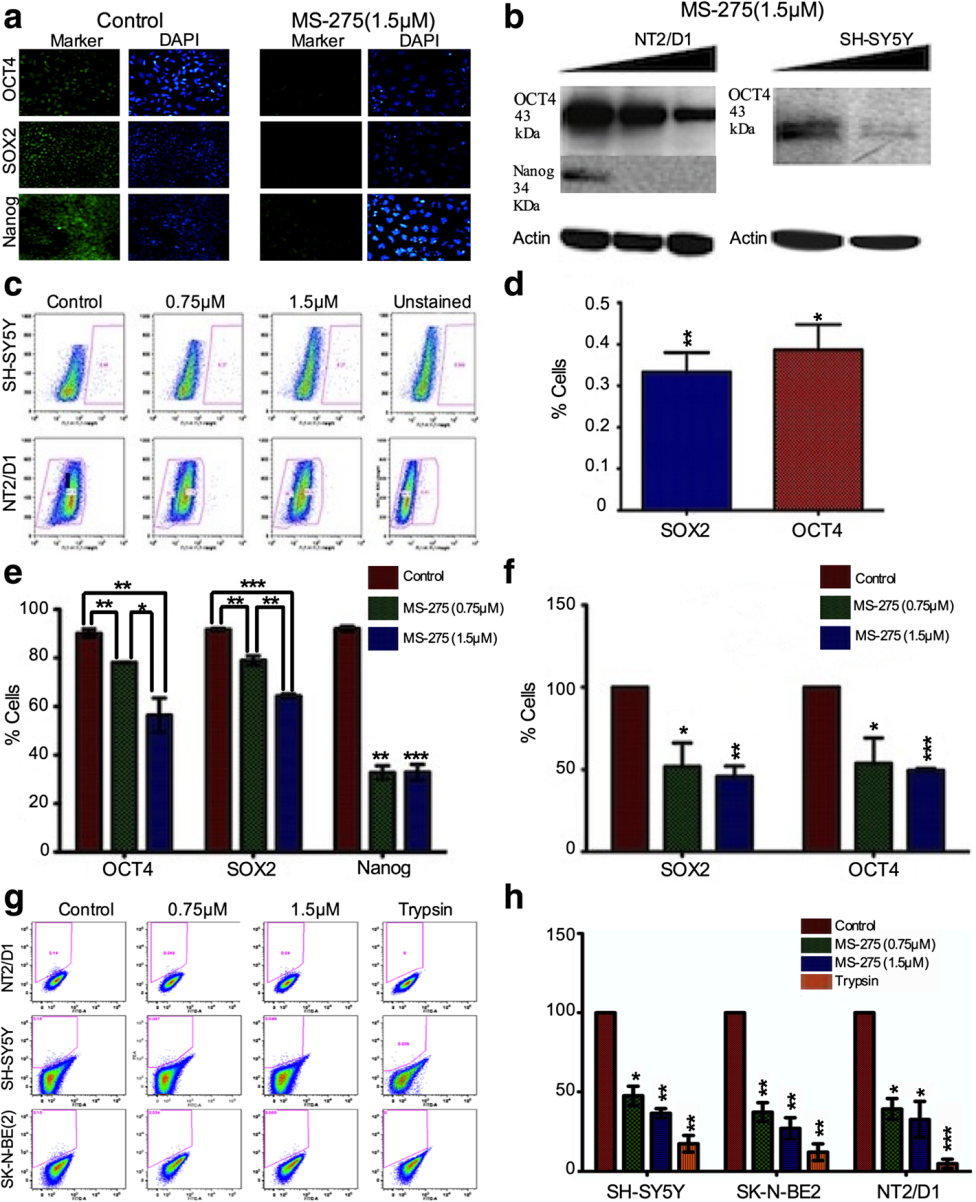
MS-275 treatment decreased expression of stem cell markers in NB and teratocarcinoma cells. **a** in the high OCT4, SOX2 and Nanog expressing NT2/D1 teratocarcinoma cell line, MS-275 was able to reduce nuclear expression of these stem cell markers as shown by immunofluorescence labeling. **b** Western blot analysis demonstrated a MS-275 reduction in expression of OCT4 and Nanog in NT2/D1, and OCT4 in SY5Y. **c** representative FACs profiles for OCT4 in SH-SY5Y and NT2/D1 cells. **d** calculated from FACs data in **c**. SH-SY5Y contains 0.38 ± 0.06% OCT4 positive and 0.33 ± 0.04% SOX2 positive cells. **e** MS-275 treatment significantly reduced expression of OCT4, SOX2 and Nanog in NT2/D1 at 0.75 μM (*p* = 0.0024, *p* < 0.0001 and *p* = 0.0031, respectively) and at 1.5 μM (*p* = 0.0096, *p* < 0.0001 and *p* = 0.0023, respectively). **f** when normalized to percentage of control, MS-275 treatment significantly reduced expression of OCT4 and SOX2 in SH-SY5Y at 0.75 μM (*p* = 0.0480 and *p* = 0.0391, respectively) and 1.5 μM (*p* = 0.0003 and *p* = 0.0066, respectively). **g** representative FACS profile for ABCG2 staining in SH-SY5Y and SK-N-BE(2) NB cell lines. **h** ABCG2 expression significantly decreases following MS-275 treatment in SH-SY5Y, SK-N-BE(2) and NT2/D1 cells at 0.75 μM (*p* = 0.0111, *p* = 0.0131 and *p* = 0.0086, respectively) and at 1.5 μM (*p* = 0.027, *p* = 0.0022 and *p* = 0.0084, respectively). Trypsin cleavage of cell surface ABCG2 was used as a negative control

**Fig. 13:**
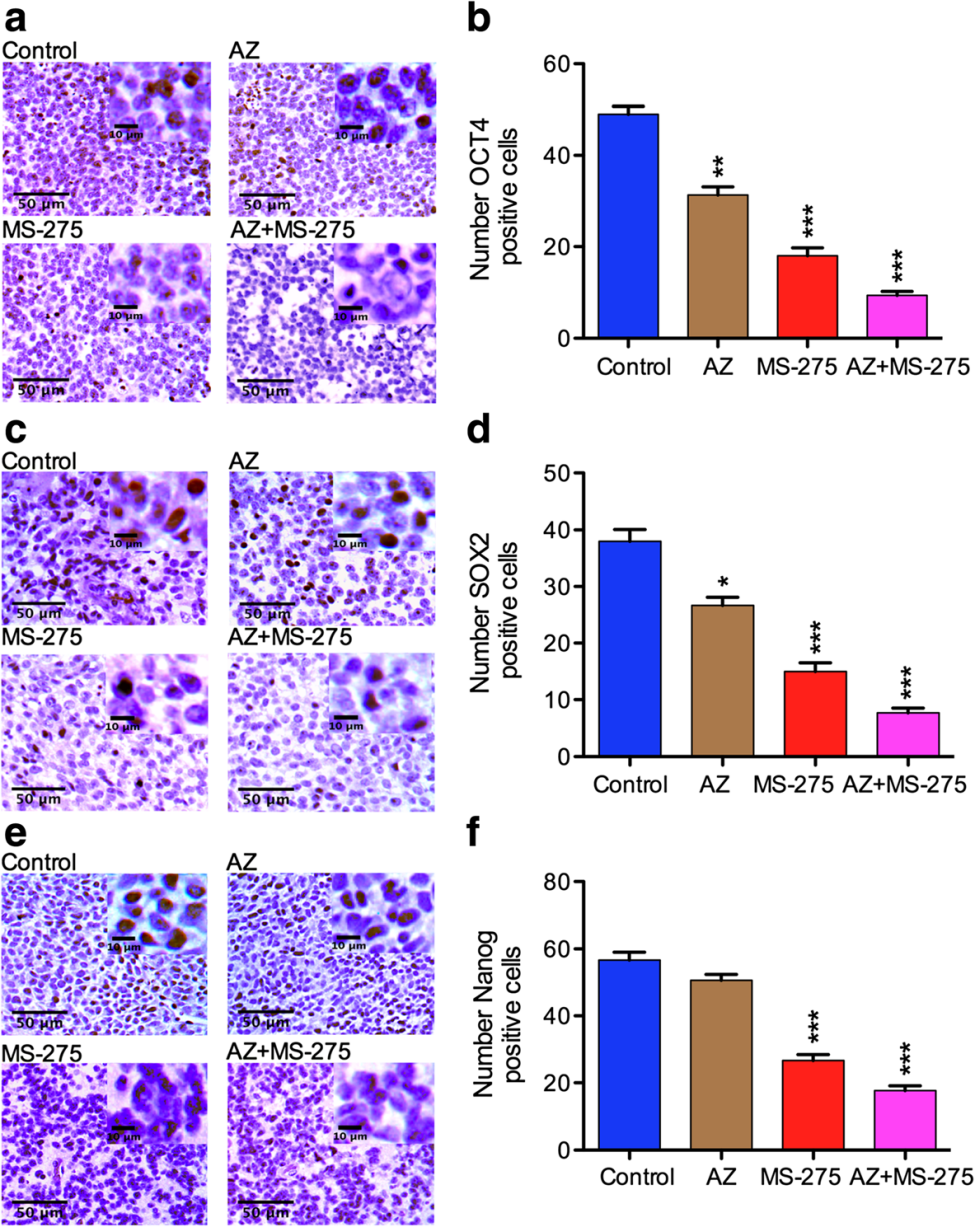
AZ and/or MS-275 treatments reduced the expression of stem cell markers in NB xenografts. **a**-**b** present IHC staining (x20 and x40) for OCT4 cell localization and number of OCT4 positive cells, **c**-**d** SOX2 cell localization and number of SOX2 positive cells, and **e**-**f** Nanog cell localization and number of Nanog positive cells after 14 days treatment with AZ, MS-275 and AZ + MS-275 compared to untreated group in SH-SY5Y xenografts. The number of OCT4 positive cells was reduced after treatment with AZ by 37 ± 0.35% (*p* < 0.05), MS-275 by 63 ± 0.85% (*p* < 0.001) and AZ + MS-275 by 82 ± 0.45% (*p* < 0.001). The number of SOX2 positive cells was reduced in AZ by 32 ± 0.60% (*p* = 0.01), MS-275 by 61 ± 0.5% (*p* = 0.0009) and AZ + MS-275 by 82 ± 0.46% (*p* = 0.0002). The number of Nanog positive cells was reduced in AZ by 11 ± 0.60% (*p* > 0.05), MS-275 by 54 ± 0.45% (*p* = 0.0005) and AZ + MS-275 by 70 ± 0.76% (*p* = 0.0002)

Our serial heterotransplantation results suggested that the CSC fraction could be targeted by a potentiated effect of AZ + MS-275. In parallel studies, we had examined the effect of MS-275 on another MYCN amplified NB cell line, SK-N-BE(2), and in comparison to the teratocarcinoma cell line, NT2 with a prevalent stem cell phenotype. MS-275 significantly inhibited clonogenic potential of SH-SY5Y and SK-N-BE(2) cells (Fig. 10a-c). Here we first established an effective concentration range for abrogating clonogenicity in methycellulose, the reduction in the presumptive tumor initiating SP cell fraction (Fig. 11a-b), compared MS-275 with TSA and found MS-275 to be more potent (Fig. 11c-d) while cisplatin (CDDP) was ineffective and reduction in expression of stem cell markers OCT4 and SOX2 by FACs and western blot analysis (Fig. 12a-h). These studies established the ability of MS-275 to reduce the tumor initiating potential of NB MYCN NB amplified cell lines. However, to expand upon these studies using immunophenotyping to determine the role of specific stemness markers (OCT4, SOX2, Nanog), we applied IHC to our SH-SY5Y xenografts after AZ, MS-275 and AZ + MS-275 treatments. We found that the AZ + MS-275 treatment produced the highest reduction in OCT4 expression, number of SOX2 positive cells, and Nanog immunopositive cells (Table 9; Fig. 13a-f). It is interesting to note that OCT4 and SOX2 expressions were most affected by AZ treatment, and relative to Nanog, proportionally greatest after AZ + MS-275 treatment, again supporting the idea of AZ potentiation of MS-275.

**Table 9:** Percentage of OCT4, SOX2 and Nanog positive cells values of SH-SY5Y tumors

Stem cell marker	Treatment modality	% Expression of positive cells (*p* value)
OCT4	AZ	63 ± 0.35 (*p* < 0.05)
	MS-275	37 ± 0.85 (*p* < 0.001)
	AZ + MS-275	18 ± 0.45 (*p* < 0.05)
SOX2	AZ	68 ± 0.60 (*p* < 0.01)
	MS-275	39 ± 0.50 (*p* < 0.009)
	AZ + MS-275	18 ± 0.46 (*p* < 0.002)
Nanog	AZ	89 ± 0.60 (*p* < 0.01)
	MS-275	46 ± 0.45 (*p* < 0.01)
	AZ + MS-275	30 ± 0.76 (*p* < 0.01)

Table 9 shows the percentage of SH-SY5Y tumors by AZ (40 mg/kg), MS-275 (20 mg/kg) and AZ + MS-275 (40 + 20 mg/kg) treatments (14D)

## Discussion

HDACis are currently being evaluated in cancer clinical trials including NB with still promising results [32]. Whether these like SAHA and MS-275 could become routinely administered is currently undecided. However, little has been done to determine if these could be potentiated with other approved drugs and in particular drugs like AZ which can be repurposed based on sound reasoning given knowledge about pH regulation in tumor cells. We took this latter approach and now report that AZ, MS-275 and especially the AZ + MS-275 combination inhibited migration, in vitro growth, induced cell cycle arrest and apoptosis of NB SH-SY5Y. In addition, the combination markedly inhibited tumor growth in vivo, reduced tumorigenicity and expression of mitosis, proliferative, HIF1-α and CAIX markers in NB SH-SY5Y xenografts. Importantly, we provide additional evidence that MS-275, at nanomolar concentrations, significantly reduced the tumor initiating cell fraction in NB SH-SY5Y and SK-N-BE. The significant reduction in initial tumorigenicity and subsequent serial heterotransplantation suggests either potential elimination or reprogramming of NB tumor initiating cells. Moreover, stemness genes (OCT4, SOX2 and Nanog) were found to be significantly down-regulated after MS-275 and the effect was enhanced by AZ + MS-275 treatment.

MS-275 has been previously shown to induce a potent G1 cell cycle arrest in NB studies [33, 34]. We confirmed this key G1 cell cycle arrest and provided evidence that dysregulation of the G1 entry checkpoint in NB is likely due to Cyclin D1 overexpression [34]. Cell cycle inhibitors that modulate cyclinD/CDK4 complex are important in G1 cell cycle arrest [8, 34]. Cyclin D1 and CDK4 knockdown results in proliferation inhibition, G1 cell cycle arrest and neuronal differentiation [35]. In this study we show that MS-275 treatment significantly reduced the expression of cyclin D1 and CDK4 relative to controls. It is not clear whether this reduction results from a direct effect of MS-275 or involves a more downstream mechanism. It has been shown that HDACi can induce the p21 cell cycle inhibitor [36]. Similarly, we found that p21 and p27 were upregulated with MS-275 treatment. Interestingly, we observed a dramatic increase in the expression of p16 CDKi. Deregulation of p16 is a common finding in a variety of neoplasms [37], and HDACi have been found to induce p16 in certain types of cancer such as colon carcinoma [38]. Induction of multiple cell cycle inhibitors would be predicted to strongly block cell cycle progression.

MS-275 induces apoptosis through different mechanisms including induction of oxidative stress, the intrinsic and extrinsic pathways of apoptosis [39]. It has been shown by Muhlethaler-Mottet [37] that inducing the intrinsic pathway of apoptosis is the most common mechanism by which HDACi such as TSA, SAHA and NaB induce apoptosis [40]. Helminthosporium carbonum (HC)-toxin (a natural HDACi) has been shown to decrease the expression of anti-apoptotic BCL2 in NB [41]. We found that the BCL-2/BAX ratio was significantly decreased by MS-275 treatment, indicating induction of the intrinsic pathway of apoptosis. BCL-2/BAX ratio also serves as a predictor of drug efficacy and cancer invasiveness [42].

We surmised that targeted inhibition of CAs by AZ could interfere with the hypoxia induced HIF1-α mediated regulation of tumor cell pH homeostasis with likely consequences to other HIF1-α regulated processes required for tumor cell growth, progression, and survival [43]. Since HDACi, such as MS-275 and SAHA, also target HIF1-α activity (e.g. translation [42]) the combination might produce a synergistic effect by blocking the ability of tumor cells to overcome hypoxic stress induced apoptosis [8, 44, 45]. Since the hypoxic niche favors localization of CSCs and their growth and survival [21], our results suggest that CSCs are targeted by the AZ + HDACi combination.

Previous studies have shown that HDAC inhibitors affect migration capacity of tumor cells [46]. The combination of trichostatin A (HDACi) and decitabine effectively decreased migration capacity of ovarian cancer cell line SKOV3 [47]. MS-275 treatment reduced migration capacity of leukemia cells [48]. Epigenetic modifications by HDACi play a key role in regulating the expression of proteins that promote or suppress tumor cell migration [49]. Tumor cell migration capacity is also enhanced by activation of the HIF1-α pathway [50], which in turn regulates CA activity. Therefore, CA inhibitors could decrease migration capacity of tumor cells. Invasion and migration are key components of the metastatic process. Here we show that the AZ + MS-275 combination significantly affected tumor cell migration using a wound healing assay concomitant with effects on growth and tumor cell survival. Thus the wound healing assay has limitations but effects on confluent cultures may predict subsequent in vivo results.

Our observations of a potentiated anti-tumor effect by AZ + MS-275 using the different assays questioned whether the effects were synergistic. In fact simple interpretations of the data might deduce synergism with certain parameters and additively with others. We therefore undertook to analyze the effects by CDI analysis and found that in monolayer cultures the combination was antagonistic at all concentrations on NSC6539 cells and additive on NSC6562 cells at concentrations above 20 μM AZ. On NB tumor cell line SH-SY5Y the combination was additive at 40 μM and 80 μM of AZ and synergistic at 160 μM of AZ. Since the combination was additive or antagonistic at all concentrations on neural stem cells, while additive or synergistic above IC50 on SH-SY5Y cells, CDI values indicate that the combination of acetazolamide and MS-275 was specifically cytotoxic on tumor cells. In fact, further in vivo results support the notion that the AZ + MS-275 combination was indeed potently cytotoxic for NB tumor cells.

Metastasis is a major problem in advanced stage NB. As indicated we found that the AZ + MS-275 combination significantly decreased migration of the SH-SY5Y tumor cells. We asked whether a key molecular contributor to the metastatic phenotype, CAIX, which has been well documented to play a role in tumor development and metastasis, was affected [51]. CAIX expression was found to be dramatically decreased by AZ + MS-275, suggesting that this combination treatment might indeed block metastatic behavior. CAIX is induced by HIF1-α, and HIF1-α knockdown significantly decreased proliferation, migration and invasiveness of NB cell lines [50, 52]. HIF1-α expression is regulated by PI3K/AKT signaling, which is blocked by HDAC inhibition [53–55]. Similar inhibitory effect on the expression of the hypoxia mediated axis in breast cancer cells has been reported for MS-275 [56]. Here, we showed that the AZ + MS-275 combination was most effective in coordinately reducing the number of HIF1-α and CAIX positive cells, correlating with a significant reduction in tumorigenic and likely metastatic potential. Taken together our findings indicate the AZ + MS-275 treatment regimen could interfere with NB metastasis at multiple levels.

**Fig. 14:**
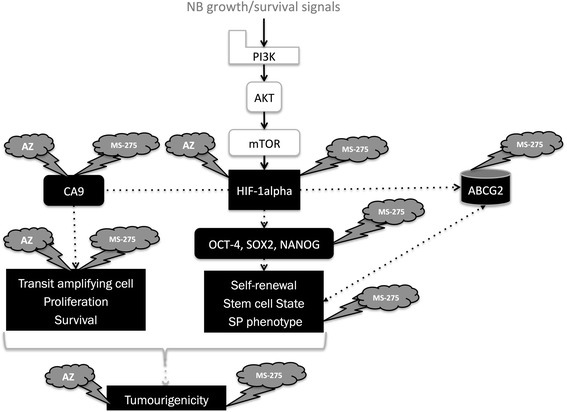
Schema depicting a potential mechanism of AZ and/or MS-275 treatments in NB cells. As illustrated, AZ, MS-275 and AZ + MS-275 treatments target survival pathways in NB cells. Alterations in cell cycle, and the HIF1-α/CAIX axis by AZ, MS-275 and AZ + MS-275 could affect the NB cell transit amplifying cell population, and its proliferation and survival. Importantly, these treatments could additionally target the CSC in NB by perturbation of self-renewal potential, stem cell state and the SP phenotype. The pan-inhibition of these pathways and critical components would ultimately decrease the tumorigenic potential of NB cells and lead to elimination of the tumor cells

MS-275 has recently been determined to be an inhibitor of the mTOR pathway by upstream modulation of AKT [55]. The mTOR pathway has also been associated with the regulation and modulation of HIF1-α, which can modulate and regulate CSCs and the stem cell phenotype [57]. Indeed, we previously showed that hypoxia induced HIF1-α signaling enhances the CSC phenotype in NB side populations (SP) [58]. CSCs are enriched in the SP fraction, a subpopulation defined by the ability to exclude the DNA-binding Hoechst 33342 dye [59, 60]. These SP cells in NB express high levels of stem cell markers, show increased tumorigenicity, and expand under hypoxia [56]. HDACi have been shown to modulate the HIF1-α mediated pathway by targeting HIF1-α towards proteosomal degradation and by repressing transactivation [61, 62]. While the modulation of AKT, mTOR and the HIF mediated pathways by HDACi is not yet fully characterized, it offers mechanistic insight showing the multiple targets for how MS-275 could be targeting CSCs (schematically shown in Fig. 14). Commonalities in signaling and gene expression has been found between normal stem cells and CSC; as such, drugs targeting normal stem cells should be investigated for their potential ability to deplete the CSC compartment.

Cancers with high percentages of cells expressing the stemness markers, OCT4 and Nanog, have been associated with prognostically poor phenotypes [63]. In this study, we showed that the combination of AZ + MS-275 significantly decreased the number of OCT4, Nanog and SOX2 positive cells in NB xenografts. In addition, we found that treatment with MS-275 significantly reduced the percentage of cells expressing OCT4, SOX2 and Nanog in NB and in a high stemness phenotype teratocarcinoma cell line. Decrease in expression of these stem cell markers in teratocarcinoma is associated with differentiation and loss of the stem cell phenotype. We also found MS-275 treatment reduced the expression of these stemness related genes in the SP fraction, taken as another means of identifying a fraction containing CSCs. NB SP cells expel Hoechst 33342 primarily by means of the ATP-dependent drug pump ABCG2 [58–60]. ABCG2 expression is regulated by AKT/PI3K signaling pathway, which HDACi is known to inhibit [64].

Finally, it is significant that MYCN non-amplified SH-SY5Y and MYCN amplified SK-N-BE(2) cell line SP fractions were equally targeted by MS-275 treatment. This critical result indicated that MS-275 could target MYCN amplified NB tumors, a group associated with the most aggressive NB phenotype and poor prognosis [1]. Cortes et al. has recently shown that actinomycin D (a member of the actinomycine family) could synergistically potentiate the efficacy of the histone deacetylase inhibitor, SAHA in NB clinical trials. In addition, the combination of actinomycin D with SAHA could inhibit tumor growth in SK-N-JD NB-xenografts [65]. While MS-275 or other HDACi such as SAHA are currently in clinical trials as promising anticancer therapeutics, however they still have limitations as single agents [66]. Therefore, a combination of a HDACi with a CA inhibitor affecting pH homeostasis, may constitute a more effective alternative therapeutic option, as we have reported in other types of cancer [17, 20] and as shown here for NB. We surmise that concomitant compromise of pH homeostasis drives tumor cells into a homeostatic crisis difficult to surmount. Since AZ has been in clinical use over a long period and the pharmacokinetics and side effects are well known and managed [67], it could be readily combined with HDACi already being evaluated in clinical trials for NB.

## Conclusions

We present convincing evidence suggesting that the AZ + MS-275 combination is likely targeting a substantial portion of NB tumor cells in vivo and in particular the CSC population in NB. This finding increases its value in terms of possible management of NB tumor progression and metastasis using two drugs with known parameters. Since CAIX expression was markedly reduced or abrogated, we propose that AZ + MS-275 should be considered for treatment of the most aggressive NB cases and could find utility for other stages and cases where metastatic progression is predictable from molecular genetic and expression analyses.

## Abbreviations

CAs: Carbonic anhydrases
HDAC: Histone deacetylases
HDACi: Histone deacetylases inhibitor
MS-275: Pyridylmethyl-N-{4-[(2-aminophenyl)-carbamoyl]-benzyl}-carbamate
NB: Neuroblastoma
